# Structure of NO16, a marine non-tailed vibriophage with an unusual symmetry-mismatched vertex arrangement

**DOI:** 10.1371/journal.ppat.1014525

**Published:** 2026-09-08

**Authors:** Sara Otaegi-Ugartemendia, Gabriela N. Condezo, Marta Martínez, Panos G. Kalatzis, Mathias Middelboe, Carmen San Martín

**Affiliations:** 1 Department of Macromolecular Structures, Centro Nacional de Biotecnología (CNB-CSIC), Madrid, Spain; 2 Escuela de Doctorado, Universidad Autónoma de Madrid, Madrid, Spain; 3 Globe Institute, Center for Evolutionary Hologenomics, University of Copenhagen, Copenhagen, Denmark; 4 Department of Biology, Marine Biological Section, University of Copenhagen, Copenhagen, Denmark; 5 HADAL & Nordcee, Department of Biology, University of Southern Denmark, Odense, Denmark; Pirbright Institute, UNITED KINGDOM OF GREAT BRITAIN AND NORTHERN IRELAND

## Abstract

Non-tailed phages remain underexplored in marine environments, as tailed phages have long dominated sequence and culture collections. Yet recent surveys suggest that non-tailed phages may be more abundant and have distinct impacts on microbial mortality and gene transfer. Here, we solve the structure of *Vibrio anguillarum* bacteriophage NO16, one of the simplest members of the *Varidnaviria* realm. Mass spectrometry detected at least nine different proteins in the virion, which has a *pseudo*T = 21 capsid similar to that of related double jelly roll (DJR) phages, but differs in the organization of minor capsid proteins, particularly those mediating membrane-capsid contacts. The DJR major capsid protein GP19 is stabilized by strong electrostatic interactions between monomers, and possibly by a cation at its base, as seen in corticovirus PM2. Localized reconstruction revealed a symmetry mismatch at the vertex, where two trimeric GP13 spikes attach to the pentameric GP14 penton base. GP13 carbohydrate-binding sites and predicted glycosylase activity point to a role in host entry. Using structural and functional predictions for its entire proteome, we propose a complete atlas of the NO16 infectious cycle.

## Author summary

Bacteriophages are among the most abundant biological entities in the oceans. While most known phages use a tail to recognize and infect bacteria, simpler non-tailed phages may be just as important in marine ecosystems. NO16 is one such virus and infects *Vibrio anguillarum*, a bacterium that causes disease in fish farms. Here, we determined the three-dimensional structure of NO16 and identified the proteins that build the virus particle. We found that NO16 is one of the simplest known members of an ancient viral lineage found across all domains of life. Despite its simplicity, it contains an unusual arrangement of vertex proteins that likely recognize sugars on the bacterial surface and help the virus enter its host. By combining structural biology with protein function predictions, we also reconstructed the likely steps of the NO16 infectious cycle. Our findings provide new insight into this overlooked group of marine viruses and their evolution.

## Introduction

While tailed bacteriophages have traditionally been considered the most abundant viruses in the marine environment, recent surveys on marine samples suggest that non-tailed phages may actually exceed tailed phages in abundance and diversity [1–4]. Most non-tailed phages are members of the *Varidnaviria* realm (formerly Double Jelly Roll lineage). These DNA viruses encode major capsid proteins (MCP) folding as a double jelly roll (formed by eight β-sheets in each sandwich) perpendicular to the capsid surface [3,5,6]. DJR lineage members include DNA viruses infecting hosts in the three domains of life: bacteria, archaea, and eukarya.

Overall, few bacteria-infecting viruses from the DJR lineage have been structurally studied compared to tailed bacteriophages. Non-tailed bacteriophages may be difficult to grow and purify in the laboratory, at least using standard phage purification protocols [7]. Only six non-tailed phage structures have been studied so far: PM2, FLiP, and ΦCjT23, which present (*pseudo) p*T = 21 triangulation capsids [8–10]; and PRD1, Bam35, and PR772, with *p*T = 25 capsids [11–13]. Among them, only FLiP, ΦCjT2, PRD1, and PR772 capsids have been solved at resolution better than 4 Å. In PM2, high-resolution information is available only for the MCP. This model was subsequently combined with lower resolution X-ray diffraction data for the whole virion [10].

*Varidnaviria* phages with available structural data are diverse in terms of host cell wall composition, genome type, and living environment. All of them infect Gram-negative bacteria, except Bam35, which infects Gram-positive *Bacillus thuringiensis.* The most common genome form is the linear dsDNA genome, shared by PRD1, PR772, and Bam35; PM2 has a circular dsDNA genome, while ΦCjT23 and FLiP have circular ssDNA genomes. These phages have been found in soil, the intestinal tract, and marine environments, where non-tailed phages are particularly abundant [2]. A newly identified non-tailed bacteriophage family, *Autolykiviridae,* has recently been classified in the *Varidnaviria* realm. Autolykiviruses belong to the order *Vinavirales* and are highly abundant, primarily lytic phages that infect marine *Vibrio* species [2]. Notably, non-tailed bacteriophages include most of the prophages that infect *Vibrio* [4].

Bacteriophage NO16, also known as *Elsinorevirus NO16* under the current ICTV binomial nomenclature [14], is a dsDNA vibriophage isolated from *Vibrio anguillarum* [15]. *V. anguillarum* is a pathogenic bacterium producing vibriosis, a mortal haemorrhagic septicemic disease affecting both cultured fish and shellfish, and causing significant losses in aquaculture industries [16]. NO16 has recently been classified into a new family named *Asemoviridae*, in the *Vinavirales* order [14,17]. The global geographic distribution of this viral family and its widespread occurrence as a prophage across >80% of the most prevalent *Vibrio* species underscores the significance of DJR phages. NO16 and NO16-like phages exhibit a temperate life cycle, enabling their integration into the genome of their *Vibrio* host and spontaneous induction. Lysogenized hosts are more virulent and better biofilm formers than their naive counterparts, indicating that NO16 impacts host pathogenicity [17].

So far, no structure has been published for any member of this new family, nor any other non-tailed vibriophage, in spite of their abundance and critical ecological role. Here, we report the first structure of a non-tailed vibriophage, one of the simplest members of the DJR lineage. Moreover, we solve the penton/spike symmetry mismatch at the vertex. Using structure and sequence homology search, we build a complete structural atlas of the NO16 infectious cycle.

## Results

### Optimized purification protocol enables high-resolution structure determination of NO16 bacteriophage

**Fig 1:**
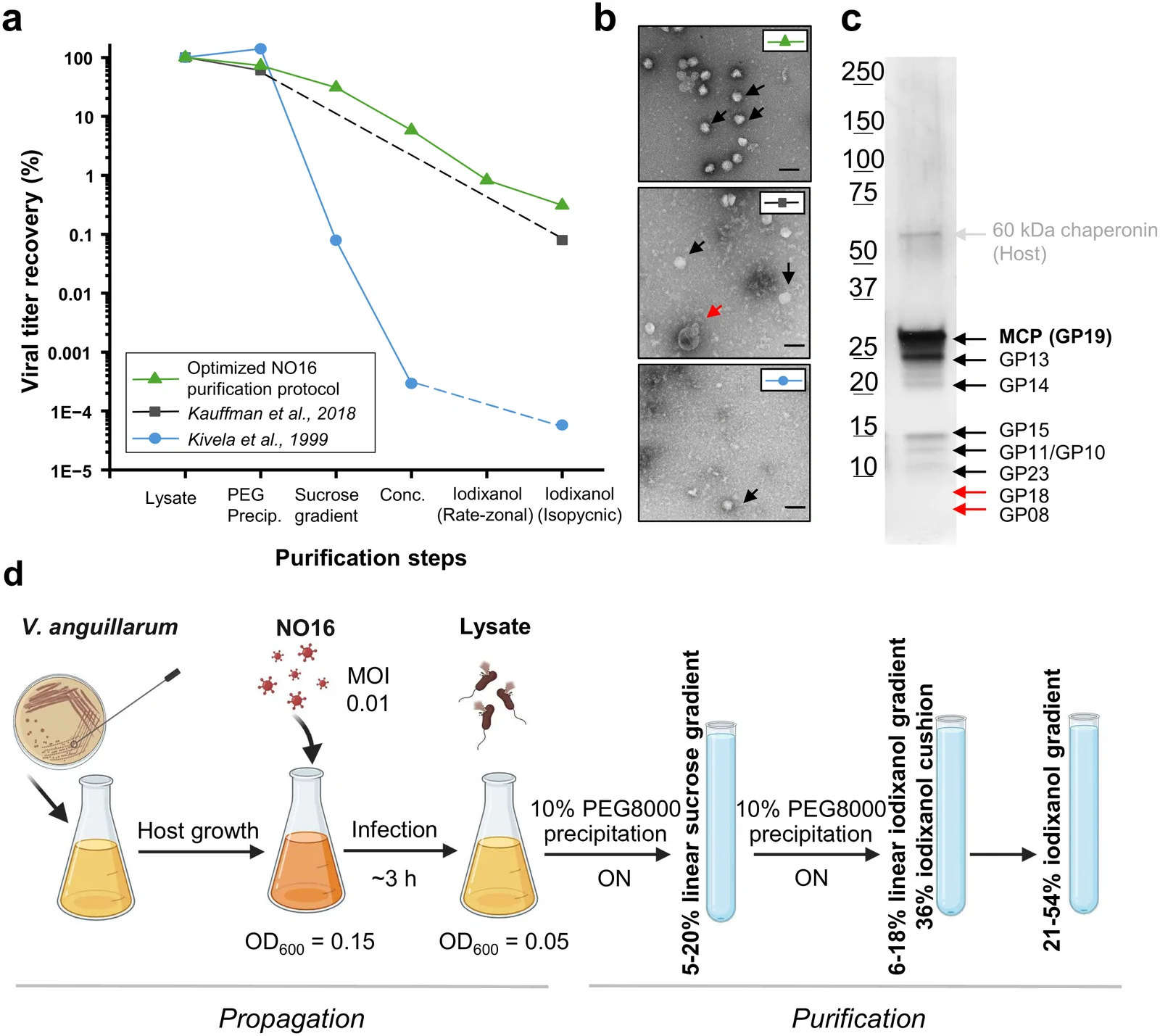
Optimization of the NO16 purification. **(a)** Viral titer recovery in each purification step in tested protocols. Dashed lines indicate intermediate steps absent in the protocol. *Conc*. stands for concentration, which was by differential ultracentrifugation in *Kivela et al., 1999* and by PEG precipitation in the optimized protocol. **(b)** Negative staining micrographs of the sample at the last purification step. Black arrows indicate NO16 particles; red arrows indicate host contaminants. Scale bars represent 100 nm. **(c)** SDS-PAGE analysis of NO16 particles purified by the optimized protocol. Tentative identity assignments to each band are based on protein molecular weights. Red arrows indicate the estimated position for non-visible bands (Table 1, S1b File). The grey arrow indicates the host 60 kDa chaperonin band, confirmed by LC-MS/MS (S1a File). **(d)** Schematic representation of the propagation and purification workflow for the optimized protocol. OD_600_, optical density values at 600 nm wavelength. MOI, multiplicity of infection. ON, overnight. Created in BioRender. https://BioRender.com/7twycnv.

Samples suitable for cryo-EM require a high concentration of particles, homogeneity, and structural integrity. We tested previously reported protocols for the propagation and purification of non-tailed phages [2,18,19] and adapted them to obtain a suitable NO16 sample for cryo-EM. The vibriophage purification protocol reported by *Kauffman et al.* [2], based on ultracentrifugation in a single isopycnic 20-54% iodixanol gradient, yielded a reasonable virus recovery, but was affected by contamination with host membranes (Fig 1a, 1b). Then, we tested the purification protocol used for the corticovirus PM2, based on an additional sucrose gradient and differential ultracentrifugation step for concentration before the iodixanol gradient [19]. Although the double gradient removed the host membranes, the decay of viral titer was pronounced at each purification step, particularly during ultracentrifugation concentration (Fig 1a, 1b). Additionally, a contaminant co-migrated with the virus in the final gradient, identified by liquid chromatography coupled to tandem mass spectrometry (LC-MS/MS) as the 60 kDa chaperonin of the host (S1a File).

**Table 1:** Proteins identified by LC-MS/MS in purified NO16 particles. Ordered by abundance.

UniProt accession	Description^1^	Molecular Weight (kDa)	Polypeptide length (aa)	pI	Abundance^2^	CN AU^3^ (Traced CN)	CN virion (Traced CN)	TM prediction^5^
A0A3G1SVL0	GP19	29.00	265	5.82	1.97E+10	10 (10)	600.00 (600)	none
A0A3G1SVN7	GP15	15.07	149	5.16	3.89E+09	1.97	118.48	none
A0A3G1SVM6	GP14	19.90	180	5.09	2.99E+09	1.52 (1)	91.07 (60)	none
A0A3G1SVP4	GP13	25.16	235	5.20	2.23E+09	1.13 (6/5)^4^	67.92 (72)	none
A0A3G1SVL8	GP11	14.00	127	9.39	3.56E+08	0.18	10.84	none
A0A3G1SVN4	GP18	8.74	82	4.66	2.40E+08	0.12 (3)	7.31 (180)	o58-i80
A0A3G1SVP5	GP10	14.39	128	8.93	2.38E+08	0.12	7.25	i7-29o
A0A3G1SVQ1	GP08	5.12	46	9.82	1.37E+08	0.07	4.17	o22-i41
A0A3G1SVP7	GP23	9.97	92	4.76	2.85E+07	0.01	0.87	none

^1^Protein names are derived from the locus tag ID at GenBank entry MH730557. For example, GP19 refers to the gene product from locus fNo16_0019.

^2^Abundance estimated using Proteome Discoverer software.

^3^Copy number (CN) estimation is calculated by dividing each protein abundance by GP19 abundance and multiplying by the GP19 CN observed in the structure (10 in the AU and 600 in the virion). CN determined by cryo-EM are indicated for comparison.

^4^Symmetry mismatch, 6 copies per pentameric vertex.

^5^Transmembrane (TM) prediction obtained using TMHMM 2.0 [72]. For example, o58-i80 indicates that residues 58–80 are predicted to cross the membrane from outside (‘o’) to inside (‘i’).

To improve viral recovery and remove the 60 kDa contaminant, the concentration step was changed from ultracentrifugation to polyethylene glycol (PEG) precipitation, and an additional iodixanol rate-zonal gradient was designed and included before the final isopycnic iodixanol gradient. This protocol succeeded in obtaining reasonable viral recovery and homogeneous NO16 particles (Fig 1a, 1b). The main protein band of the purified sample corresponded to the MCP size (29 kDa), confirming its purity (Fig 1c). This final protocol is detailed in Fig 1d.

LC-MS/MS of the purified virion preparation identified 9 proteins out of the 23 predicted Open Reading Frames (ORFs) encoded by the 10.5 kbp circular dsDNA genome (Table 1, S1b File) [15]. Bands consistent with the molecular weights of most of these proteins were observed in SDS-PAGE of the purified sample (Fig 1c). The structures of all nine proteins detected in the virion were predicted using AlphaFold (S1 Fig). The structure predicted for GP19, the most abundant protein in the virion (Table 1, Fig 1C), included the DJR fold (S1 Fig).

**Fig 2:**
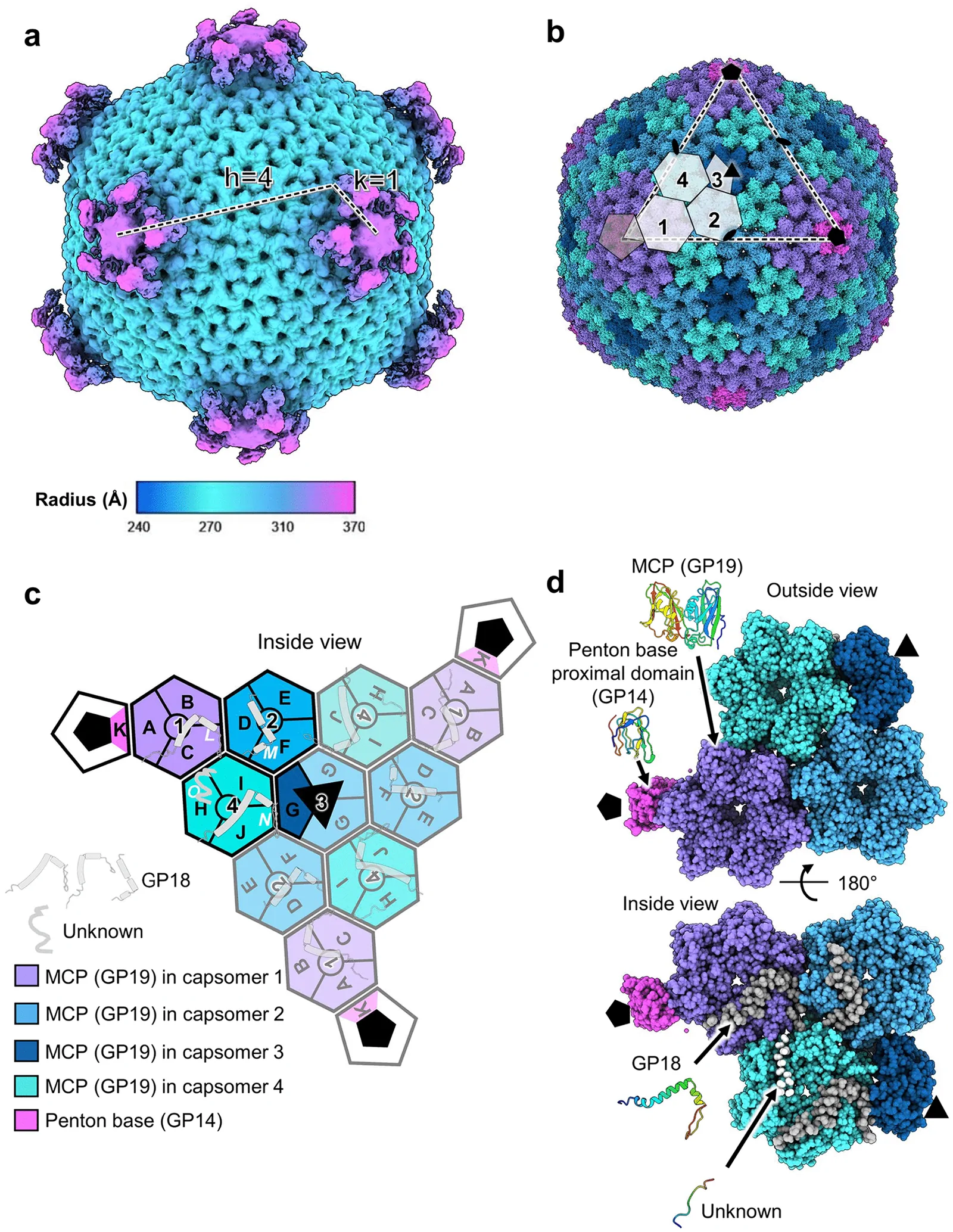
Cryo-EM structure of bacteriophage NO16. **(a)** Cryo-EM map colored by radius. Dashed black lines define h and k, lattice coordinates or displacement vectors of a pentamer relative to another pentamer used to calculate the triangulation number (T = h^2^ + hk + k^2^) [20]. **(b)** Molecular models for the proteins traced in the icosahedrally averaged map. The three MCP trimers and one MCP monomer in the asymmetric unit (AU) are numbered 1 to 4. The capsid facet is identified with dashed lines. Icosahedral symmetry axes are indicated with black symbols: fivefold (pentagon), threefold (triangle), and twofold (oval). MCP trimers are coloured according to their position in the AU. Color legend in (c). **(c)** Schematics showing the organization of one icosahedral facet, as viewed from inside the capsid. One AU is highlighted, and the others are semi-transparent. Letters A-J indicate chain identifiers for MCP monomers. The mCP GP18 chain identifiers are in white. Protein colours and shapes are indicated in the legend. **(d)** Detail of the AU as seen from outside (top) and inside (bottom). Color legend in (c). Representative protein structures are rainbow-colored, from blue (N-terminus) to red (C-terminus).

To solve the structure of bacteriophage NO16, we collected cryo-EM data and applied single particle averaging with icosahedral symmetry (S1 Table, S2a Fig). The resulting cryo-EM map, at an overall 3.3 Å resolution, showed that the NO16 capsid has a triangulation number T = 21d [20] (Figs 2a, S2b, S2c). This triangulation number was previously observed in bacteriophages PM2, FLiP, and ΦCjT23 [8–10], and represents the smallest reported triangulation number in the DJR lineage. NO16 bacteriophage particles have diameters of 57 nm (facet-to-facet) and 70 nm (vertex-to-vertex) (Fig 2a).

We traced three proteins in the icosahedrally averaged map (S2, S3 Tables): the MCP (GP19), penton base protein (GP14), and minor capsid protein (mCP) GP18, located on the inner capsid surface (Fig 2b–2d, S1 Movie). The asymmetric unit (AU) is composed of 10 MCP monomers, organized into three trimers plus one monomer forming the trimer centered at the icosahedral 3-fold axis, and a monomer of vertex protein (Fig 2b). As the MCP forms trimeric pseudo-hexamers, the NO16 triangulation number is best defined by pseudo-triangulation (*p*T) = 21d.

### The shortest MCP among dsDNA varidnaviruses

**Fig 3:**
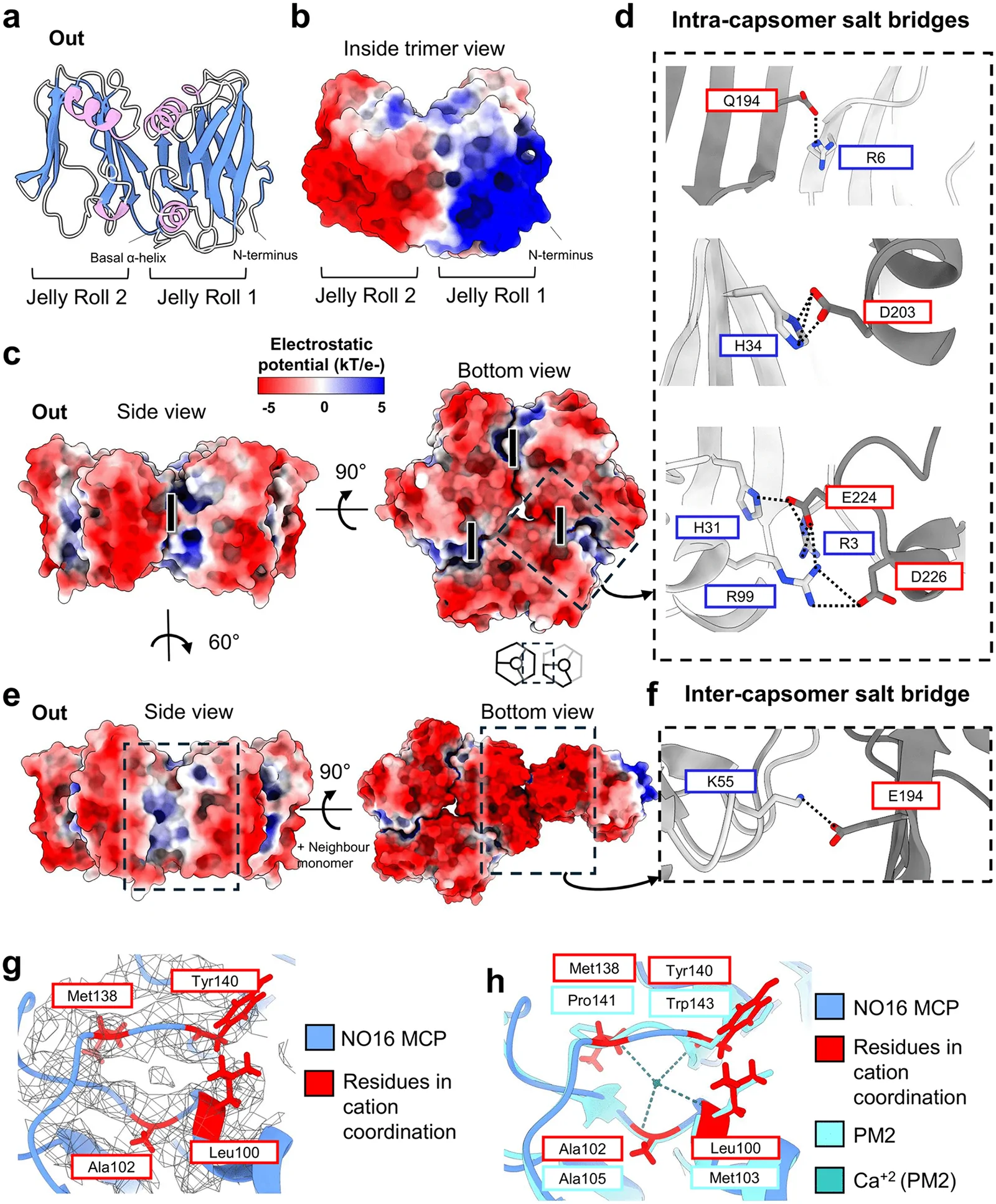
Structure of NO16 MCP, GP19. **(a)** Molecular model of the MCP monomer, with α-helices in pink and β-strands in blue. **(b)** MCP monomer surface colored by electrostatic potential, viewed from inside the trimer. **(c)** MCP trimer capsomer, from the side (left) and bottom (right), colored by surface electrostatic potential. I indicates interface between monomers. Details from the region inside the dashed square are shown in (d). **(d)** Intra-capsomer interactions between monomers, with amino acids taking part in salt bridges colored by heteroatom. Dashed lines indicate salt bridges. **(e)** MCP interface contacting neighburing capsomers indicated in dashed squares (left) and inter-capsomer contact (right), colored by surface electrostatic potential. The cartoon represents the inter-capsomer contact. Details from the interface inside the dashed square are shown in (f). **(f)** Inter-capsomer interactions between monomers, with amino acids taking part in salt bridges coloured by heteroatom. A dashed line indicates the salt bridge. **(g)** Cation coordination site at the base of the NO16 MCP with the sharpened map rendered at 4.5σ. Residues potentially involved in protein-ion interactions are indicated in squares. **(h)** Structural alignment of Ca^+2^ coordination site in PM2 and NO16 MCPs. Residues involved in the interactions are indicated in squares.

The NO16 MCP (GP19, 265 amino acids (aa) long) presents a conserved DJR fold, with two β-sandwiches separated by a small α-helix at their base and two α-helices on the outer surface (Fig 3a). The NO16 MCP is the shortest one so far reported for the dsDNA *Varidnaviria*, with only the MCP of ssDNA phage ΦCjT23 (P5, 239 aa) being shorter (S3a Fig) [9]. Structural comparison shows that NO16 MCP is most closely related to that of the corticovirus PM2 (P2, 296 aa) [10] (S3b–S3d Fig), with a DALI Z-score of 36.3. PM2 has so far served as the prototype for minimal capsid architecture among dsDNA varidnaviruses. However, the shorter length of its MCP suggests that NO16 may represent an even more reduced and potentially minimal structural framework within the dsDNA branch of the realm.

The GP19 N-terminal jelly roll (jelly roll 1) is positively charged, while the second (jelly roll 2) is negatively charged (Fig 3b). MCP monomers assemble into trimers to form the capsomers, helped by these strong complementary charges (Fig 3c). Between six and twelve salt bridges can be formed in the NO16 intra-capsomer contact surfaces (S4 Table), distributed in five unique residue-residue salt bridge interactions (Fig 3d). A similar charge distribution was observed in PM2, but not in FLiP and ΦCjT23 (S3e Fig). Indeed, PM2 MCP is stabilized by up to 10 salt bridges, double the number detected in ΦCjT23 and FLiP MCP (S5 Table). In contrast, electrostatic interactions do not play a major role in NO16 inter-capsomer contacts (Fig 3e), with only one residue-residue salt bridge observed (Fig 3f, Table S4).

In the NO16 cryo-EM maps, we observed spherical densities well above noise level (4.5σ) in chains A, E, I, and J, at the same position where a Ca^+2^ ion was observed in PM2 [10] (Figs 3g, S3f). These densities might correspond to a Ca^+2^ or a Mg^+2^ ion, since both are present in the buffer. This ion could have a role comparable to that observed in PM2, where treatments with EDTA destabilized the capsid [18], and in the absence of Ca^2+^ the infection was arrested [21]. In PM2, the Ca^2+^ ion was in coordination with residues M103, A105, P141, and W143 [10] (Fig 3h). Structural alignment revealed that in NO16 MCP, residues L100 and Y140 (corresponding to M103 and W143 in PM2) preserve the chemical environment of the Ca^2+^ binding pocket, whereas M138 (P141 in PM2) does not. Unfortunately, neither infectivity changes upon treatment with chelating agents, nor computational prediction, provided conclusive evidence for the identity of this putative ion or for a stabilizing role of divalent cations in NO16 particles (S4 Fig).

### Two trimeric spikes at the pentameric vertex in a previously unseen symmetry mismatched arrangement

Penton proteins in DJR viruses are more variable than MCPs. They often present a single jelly roll (SJR) in the penton base [3]. In some cases, this SJR is augmented by another protruding SJR, and/or by spike proteins. No penton base or spike proteins were annotated in the NO16 genome; however, two of the proteins detected in purified virions by mass spectrometry were predicted to have SJR folds: GP13 and GP14 (S1 Fig). The N-terminal domain of GP14 (residues 2-89 aa) was identified and traced in the icosahedrally averaged map, forming a SJR buried in the gap left by the peripentonal MCP capsomers (Fig 2d). Density consistent with an additional predicted SJR formed by the distal C-terminal domain was observed, but its lower resolution did not allow tracing the rest of the protein (S2c Fig). Further, additional density formed five petals around the distal part of the vertex. The lower signal of this structural feature suggested the presence of flexible or symmetry-mismatched elements at the vertex (S2c Fig).

**Fig 4:**
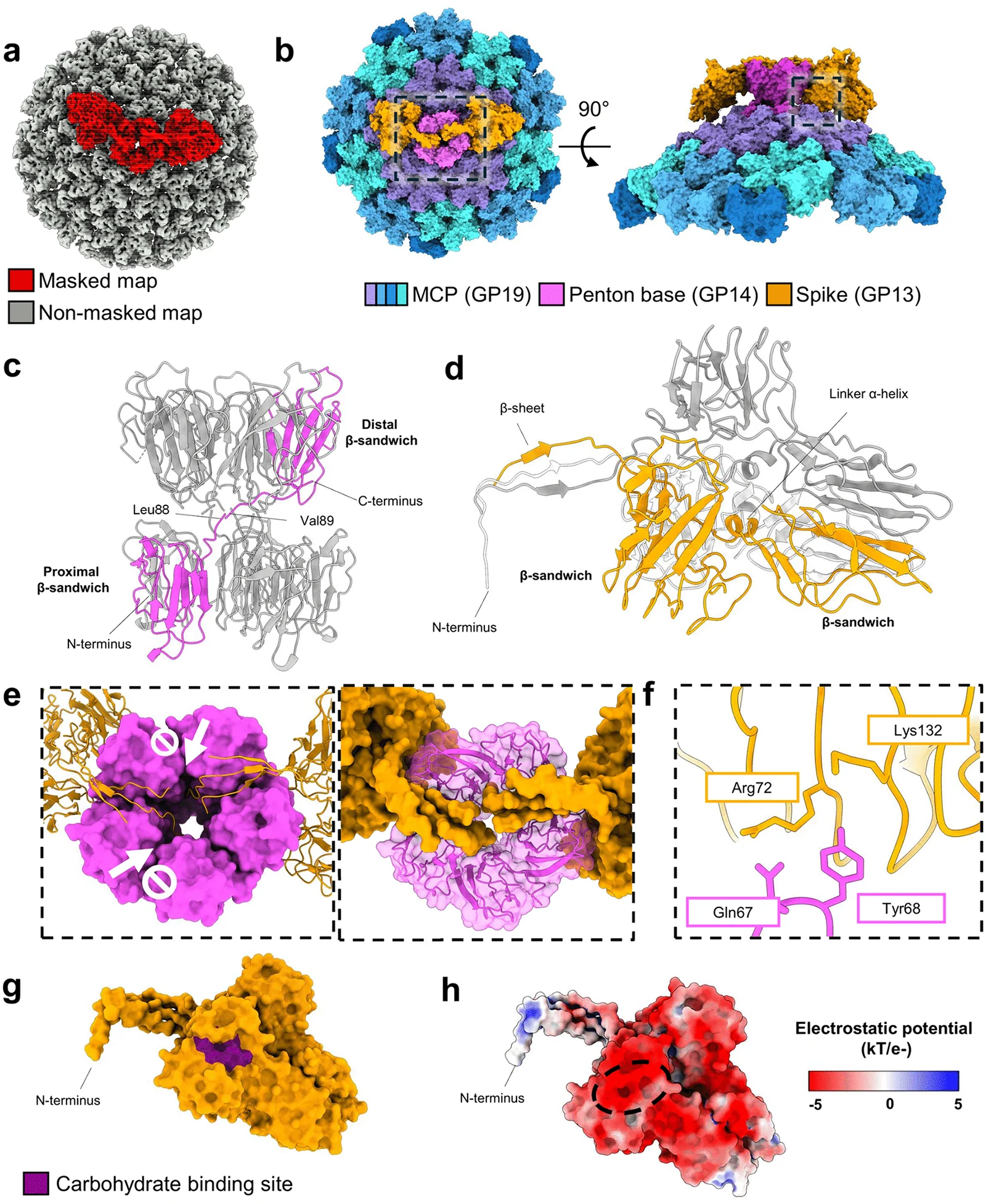
Structure of the vertex region. **(a)** Overlap of localized reconstruction maps generated without using any mask (grey) and using a localized mask of the distal penton domain surrounded by two petals (red). **(b)** Molecular model for the vertex region. The dashed square in the left panel is detailed in (e), and the dashed square in the right panel is detailed in (f). **(c)** Penton base GP14 structure, with a monomer in pink and the rest of the monomers in grey. **(d)** Spike GP13 trimer structure, with one monomer in orange and the rest in grey and white. **(e)** The N-terminal stalk of GP13 anchoring the spike to the pentamer GP14 central cavity. White arrows and block symbols indicate GP14-GP14 interfaces blocked by GP13 N-termini. Proteins colored as in (b). **(f)** Contacts between spike and proximal penton base region. **(g)** Spike trimer highlighting the carbohydrate binding site. **(h)** Spike trimer colored by electrostatic potential. The dashed oval indicates the carbohydrate binding cleft.

To characterize the five petal densities around the distal domain of the GP14 pentamer, we carried out a localized reconstruction analysis of the vertex region (see Methods) (S5a–S5d Fig). Subparticle classification revealed that the best-defined vertex classes included only two opposed petals, one of them with higher density than the other (S5c Fig, red frames). This unexpected symmetry mismatch at the vertex was solved at 3.9 Å resolution in the final map (Figs 4a, S6a, S6b). In this localized reconstruction, we traced the GP14 distal domain, which showed that the protein has the conserved penton base structure of a SJR inserted in the capsid shell, and another SJR protruding from the surface of the virus particle (Fig 4b, 4c). The GP14 proximal and distal domains are connected by a flexible link (Fig 4c).

GP13, the second candidate for the vertex protein, hereafter referred to as spike protein, was identified forming a trimer on the density of each one of the two opposed petal structures (Fig 4b,4d, S1 Movie). The GP13 monomer interacting with the capsid (chain F) was better ordered than the distal monomers, suggesting that the latter, not stabilized by capsid contacts, display higher flexibility. Therefore, whereas the proximal monomer was fully modelled, flexible distal monomers were partially modelled (S6c Fig, S3 Table). The main body of GP13 is composed of two β-sandwich domains linked by a small α-helix and positioned perpendicularly to each other (Fig 4d).

The two trimeric GP13 spikes bind to the GP14 pentamer in a symmetry-mismatched manner (Fig 4b). In each spike, the N-terminal domains (residues 3-20) form an anchoring stalk composed of three small parallel β-strands (Fig 4d, 4e). The two stalks run along GP14 monomer-monomer interfaces in the pentamer. At the pentamer center, the stalk bends sharply and enters the penton base central cavity (Fig 4e). The N-terminal stalks extend to the neighboring GP14-GP14 interface, blocking the binding of an adjacent spike (Fig 4e). As a consequence, the second spike must occupy a non-adjacent, opposed position, and addition of a third spike is prevented. This observation aligned with the result of a second localized classification discriminating between presence and absence of spike at a specified position (S7 Fig). The class that fixed one spike at position 1 showed two low density spikes at positions 3 and 4, pointing to their partial occupancy, as only one of them could be filled without clashing with the other (S6d Fig). Apart from the N-terminal anchoring, Arg72 and Lys132 in the GP13 monomer closest to the capsid form hydrogen bonds with Gln67 and Tyr68 of the GP14 N-terminal SJR (Fig 4f, S4 Table). These interactions may contribute to the stabilization of the GP13 monomer adjacent to the capsid surface.

GP13 spikes are parallel to the capsid, differing in orientation from those in related viruses such as PRD1 [22], STIV [23], and adenovirus [24] spikes, which protrude radially. Homology search with DALI [25] and Foldseek [26] showed structural similarity with proteins presenting carbohydrate-binding modules (CBM) (S2a, S2b File). DALI indicated that GP13 was most similar to Family 16 CBM S-layer associated multidomain endoglucanase [27], and Foldseek indicated structural similarity to Vip3Bc1 and Vip3Aa protoxin structures harboring CBMs [28, 29] (S6 Table, S2a, S2b File). The carbohydrate-binding site of GP13, located in the N-terminal β-sandwich, displays a curved surface with a cleft (Fig 4g), resembling that of its DALI homologue [27], where sugars are thought to interact with polar and acidic residues (Fig 4h). This cleft is solvent-exposed, similarly to protoxin CBMs [29]. The map resolution was not sufficient for tracing the clefts in monomers 2 and 3, consistently with the reported flexible nature of carbohydrate binding sites [29] (S6c Fig, S3 Table).

Additionally, CLEAN enzymatic function prediction software indicated that GP13, even with a low confidence level, could be a glycosylase, a hydrolase that catalyzes the cleavage of the glycosidic bond (S6 Table). Considering that vertex proteins in DJR viruses generally mediate the entry process [30, 31] and that carbohydrate binding sites are present in tail spike structures in *Caudovirales*, these observations strongly suggest that GP13 could have a role in host surface attachment, recognition, and/or hydrolysis of saccharide molecules.

### A capsid shell with minimal membrane contact

**Fig 5:**
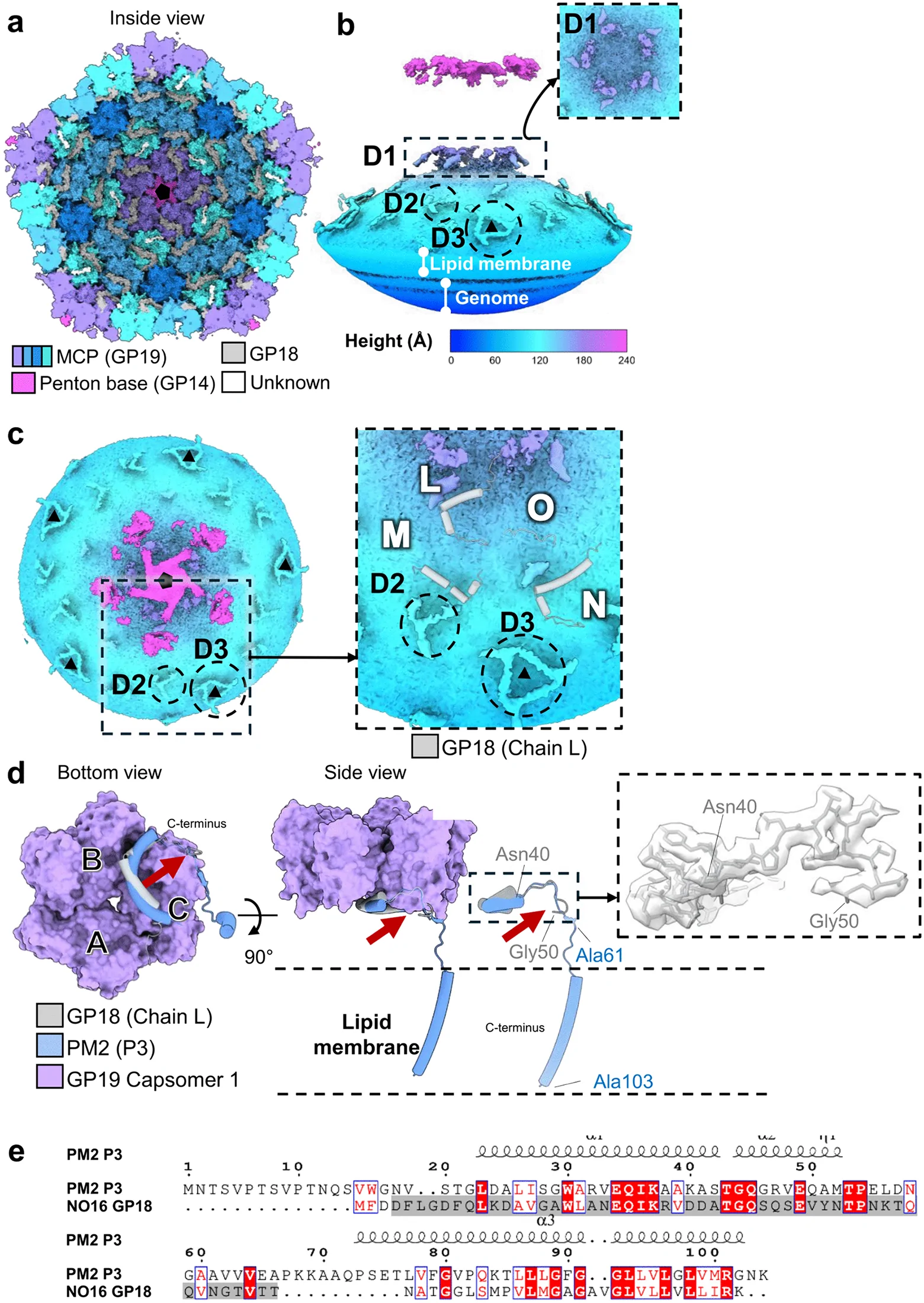
Minor capsid protein organization. **(a)** NO16 inner capsid surface organization viewed along the 5-fold axis. **(b)** Localized reconstruction colored by height along the 5-fold axis, showing non traced densities possibly corresponding to mCPs. The signal from the capsid structure was subtracted, and the predominant visible density corresponds to the internal membrane. A view of the D1 densities as seen from the capsid is shown in the top right panel. **(c)** The map in panel (b), rotated -90 degrees around the x-axis. The region inside the dashed box is zoomed on the right. **(d)** GP18 and PM2 P3 protein comparison. Capsomer 1 viewed from inside (left) and from the side (right), with NO16 GP18 and PM2 P3 proteins overlapped. The dashed box highlights a region of GP18 in chain L, along with its corresponding cryo-EM map density. **(e)** Sequence alignment of PM2 P3 and NO16 GP18 visualized by ENDscript. The traced region of NO16 GP18 (residues 4-55 aa) is highlighted in grey.

We identified GP18 as the only mCP on the inner side of the NO16 capsid (Figs 2c, 2d, 5a). Three copies of GP18 were traced per AU, located beneath capsomers 1 (chain L), 2 (chain M), and 4 (chain N) (Fig 2c). Only the N-terminal domain of GP18 appears to be well ordered (S3 Table). Residues 58-80 in the C-terminal domain are predicted to form a transmembrane region (Table 1). However, unlike in other DJR phages, no densities connecting capsid and membrane were observed (S8 Fig). GP18 exhibits some complementary charges to the MCP pockets in which it is embedded (S9a Fig) and its binding to the capsomers is energetically favored, as shown by the negative solvation-free energy (ΔiG values of -14.8, -15.2, and -19.3 for the L, M, and N chains, respectively), considerably stronger than any other interactions in the AU (S4 Table). This observation suggests that GP18 may act as a hub to help recruit capsomers during assembly. Although they could not be traced due to low resolution, other mCPs seem to be present. For example, chain O was observed next to GP18 in capsomer 4 position (Fig 2c, 2d), and modelled as a 12 residue-long ‘unknown’ chain (S3 Table), since ModelAngelo could not confidently assign this density to any sequence in the NO16 proteome.

Although the icosahedral map did not show additional densities, three main remnant densities appeared in localized reconstructions (Fig 5b). This effect can be attributed to the subtraction of the capsid signal from the subparticle projections, which enables independent refinement of the remaining features. The main density (D1) is situated beneath the 5-fold axis and mediates contacts with the lipid membrane; a second, small remnant density (D2) is located below capsomer 2; and the third and last density (D3) is positioned around the 3-fold axis (Fig 5b, 5c).

To better understand GP18 interactions with the membrane, and to assign an identity to chain O and the unmodeled remnant densities, we compared our structure with that of PM2. GP18 resembles the PM2 mCP P3 in position and protein sequence identity (39%). Indeed, P3 is a conserved protein among *Vinavirales* non-tailed bacteriophages [32]. In PM2, four copies of P3 were traced per AU, with the additional copy located below capsomer 3. In PM2, the copy of P3 located below capsomer 1 enters the membrane (S9b Fig). In contrast, we did not observe any hints of density that could correspond to the C-terminal region of NO16 GP18 under capsomer 1 interacting with the lipid membrane. Although absence of density may result from flexibility and/or non-icosahedral organization, it is notable that this absence was reproduced in the localized reconstruction maps (Fig 5c). Further, in the icosahedrally averaged map the pathway followed by the central region of GP18 clearly turns back towards the inner capsid surface and away from the membrane, unlike in PM2 (Fig 5d, red arrows). This deviation from the path towards the membrane occurs also in the GP18 copy near capsomer 4, where the traced model reaches residue Asn55, located at the beginning of the predicted transmembrane helix (Figs 5e, S9c). NO16 GP18 is shorter than PM2 P3, particularly in the region linking its two predicted α-helical regions. This shorter connection could hinder the ability of the GP18 C-terminal region to reach the lipid membrane, or to establish a stable, ordered capsid-membrane interaction.

In PM2, an additional mCP, P6, homologous to NO16 GP10, reinforces the mCP network and also contacts the inner lipid membrane (S9b Fig). P6 is located below capsomer 4 near the 2-fold axis, and is clearly visible in the central slice of the PM2 cryo-EM map [33] (S8 Fig). In NO16, although GP10 is predicted to be homologous to P6, it does not correspond to any traced proteins or remnant densities (S9d Fig).

Chain O does not align clearly with any known mCP positions in PM2 (S9c Fig). Additionally, the remnant density D1 is a feature absent in PM2, as there is no electron density below the vertex region (S8 Fig). Although the resolution of this density was not enough for identification, taking into account that the protein would necessarily be in the particle and present a transmembrane region, GP08 and GP10 are feasible candidates (Table 1, S1 Fig). D2 does not overlap with any of the PM2 mCP either, and its small, indistinct shape prevents reliable identification. In contrast, D3 is positioned near the 3-fold axis, precisely where the fourth copy of P3 is located in PM2 (S9d Fig). This positional match strongly suggests that D3 represents an additional copy of GP18.

There is little information on the mCP network for other T = 21 bacteriophages in the DJR lineage. In FLiP, the C-terminal helix of the MCP is believed to interact with the membrane [8], without the need for mCP (S8 Fig). In ΦCjT23, although densities contacting the membrane under capsomers 2 and 4 were observed, and the virus encodes P3 and P6 homologues, no mCP was traced [9]. ΦCjT23 may resemble NO16 in that it contains mCPs homologous to those of PM2, and that these mCP do not reach the membrane.

### Complete NO16 structural atlas

To elucidate putative functions of all NO16 proteins, we used AlphaFold structure prediction and considered two categories: proteins present in the virion but not solved in the cryo-EM map, and proteins encoded by NO16 but not detected in the virion.

Apart from proteins localized in the map, mass spectrometry indicated the presence of five additional proteins in the viral particle: GP15, GP08, GP11, GP10 and GP23 (Table 1). All these proteins, except GP15, have low abundance, with one or fewer estimated copies per virion (Table 1). However, many of these proteins are short and hydrophobic, which may hinder their quantification by LC-MS/MS, potentially biasing their abundances and, therefore, the estimated copy numbers.

Only the GP11 AlphaFold prediction (S1 Fig) had high enough reliability to be used for structural homology search. Both DALI and Foldseek detected strong structural homology with the endolysin encoded by *Hafnia* phage Enc34 (S6 Table, S2c, S2d File). Enc34 is a siphovirus that infects Gram-negative bacteria, including the opportunistic *Pseudomonas aeruginosa* PAO1 [34]. Endolysins are viral peptidoglycan-degrading enzymes that digest the bacterial cell wall [35], usually for release of the virus progeny by lysis of the host. Additionally, CLEAN predicts GP11 to have lysozyme activity (S6 Table).

Out of the proteins encoded by NO16 and not detected in the virion (S10 Fig), GP03, GP06, GP07, GP16, and GP21 AlphaFold predictions were submitted to homology search, as their predicted models had pTM scores above 0.75, indicating sufficiently high confidence. DALI indicated that GP03 has slight (Z-score = 9.3) structural homology to *Sulfolobus islandicus* rod-shaped virus 1 (SIRV1) virus protein ORF119, a replication initiation protein, while Foldseek did not identify significant homologues (S6 Table, S2e, S2f File). InterPro supported the possibility that GP03 may function as a replication-associated protein, part of the rolling-circle DNA synthesis machinery, with cleavage and ligase activity (IPR056906). CLEAN indicated, with low confidence, that GP03 may be a DNA ligase.

GP06 is structurally similar to transcriptional repressors (S6 Table, S2g, S2h File). In both DALI and Foldseek searches, λ phage repressor appeared as a candidate. The λ phage repressor acts as a genetic switch that enables the phage to transition to lysogeny by binding to a lytic promoter [36]. As λ phage repressor is a dimer, GP06 might also form a dimer [36], and AlphaFold ipTM scores also support this possibility (S10 Fig). Only the N-terminal region of GP07 (residues 1-54) is predicted to be ordered (S10 Fig). Homology search of this N-terminal region returned similarities to anti-clustered regularly interspaced short palindromic repeats (Anti-CRISPR) associated proteins with both DALI and Foldseek (S6 Table, S2i, S2J File). Phages use anti-CRISPR associated proteins to inhibit host CRISPR-Cas systems, which normally act as transcriptional repressors [37, 38]. The prediction indicates that the N-terminal domain of GP07 interacts with dsDNA and forms a dimer [38], suggesting that full length GP07 might also adopt a dimeric configuration (S10 Fig). For GP16, both DALI and Foldseek indicated homology to bacterial acetyltransferases (S6 Table, S2k, S2l File). Acetyltransferases encoded by bacteriophages of the lineage have been previously reported [32]. Finally, GP21 is structurally similar to the genome packaging NTPase of the DJR archaeal virus *Sulfolobus* turreted icosahedral virus 2 (STIV2) (S6 Table, S2m, S2n File), which is predicted to form hexamers [39]. AlphaFold ipTM scores for oligomers also support the prediction that GP21 is a hexamer (S10 Fig). The genome packaging ATPase is conserved throughout the *Varidnaviria* realm [17, 40], and is thought to function as a motor for energy-dependent packaging of virus genomes into capsids. InterPro predicts a helicase HerA-like domain, found in archaeal helicases and some putative helicases from bacteria. This domain becomes ordered when dsDNA is included in the AlphaFold prediction (S10 Fig). CLEAN predicts GP21 to be a site-specific deoxyribonuclease (S6 Table).

## Discussion

Sample preparation is one of the most significant bottlenecks in cryo-EM, particularly for less well-characterized specimens. The infection efficiency of NO16 varies among different populations of *Vibrio anguillarum*, even within the same strain [17]. Moreover, membrane-containing viruses are more sensitive to salinity than non-lipid containing viruses [41]. Optimization of the purification protocol for NO16 was challenging, and three density gradient steps were necessary to obtain a suitable sample for cryo-EM. Structural data on non-tailed phages remain scarce, with only four capsid structures solved at near or beyond 4 Å resolution [8, 9, 11, 12]. Moreover, despite achieving high resolution, most of the structures are incomplete, since only PRD1 and PR772 structures contain traced mCP proteins [11, 12], while PM2 mCPs were modelled in an X-ray map at 7 Å resolution [10].

### One of the simplest capsids in the *Varidnaviria* realm

The *Varidnaviria* realm contains viruses with genome sizes ranging from 10 kbp to 1,500 kbp and capsid diameters between 60 and 2,500 nm, presenting a wide range of icosahedral geometries from T = 21 up to T = 912-1,200 [42]. NO16 presents a T = 21 capsid, which is the smallest observed among realm members, and its MCP (265 aa) is the shortest reported to date among dsDNA members of the lineage, with only the ssDNA phage ΦCjT23 having a shorter MCP.

Even though the general organization of the NO16 capsid is similar to that of PM2, the organization of the inner mCP network is simpler. It is widely recognized that cementing proteins are essential for proper viral assembly and, in these viruses, membranes appear to serve as scaffolding platforms that facilitate the recruitment of capsid-forming proteins [10]. However, no ordered membrane-capsid contacts have been visualized in NO16, although tenuous density seemed to connect capsid and membrane near the vertex. This observation indicates that, even if capsid-membrane interactions are needed for assembly, they may not need to be maintained once the particle is formed. This is similar to packaging protein L1 52/55k, which is processed by adenovirus protease during maturation [43], and to scaffold proteins in *Caudovirales*, which are also removed during the transition from procapsid to mature capsids [44].

Given the structural features of the MCP and the mCP network, NO16 is simpler than PM2, and is therefore the simplest dsDNA virus of the DJR lineage, with ssDNA ØCjT23 being the simplest in the *Varidnaviria* realm. Therefore, NO16 represents a minimal prototype of the dsDNA *Varidnaviria* structural framework, and may lie near the base of the dsDNA varidnavirus evolutionary branch.

### The symmetry-mismatched vertex and its potential for host attachment

It is quite common for spike proteins to show symmetry mismatches and flexibility, as in PRD1 [22, 45] or adenovirus [24, 46, 47]. In NO16, we found two trimers of spike protein GP13 assembled in a symmetry-mismatched manner onto the GP14 pentamer. In previous studies on *Varidnaviria* bacteriophages, attempts at localized reconstruction failed to obtain enough resolution to trace the spike proteins [9, 11]. Apart from tailed phages and their herpesvirus relatives, where prominent structural features and strong cryo-EM signals have enabled the successful resolution of symmetry mismatches, this represents, to the best of our knowledge, the first time in which localized reconstruction solved the symmetry-mismatched vertex region at enough resolution to trace the spike protein.

**Fig 6:**
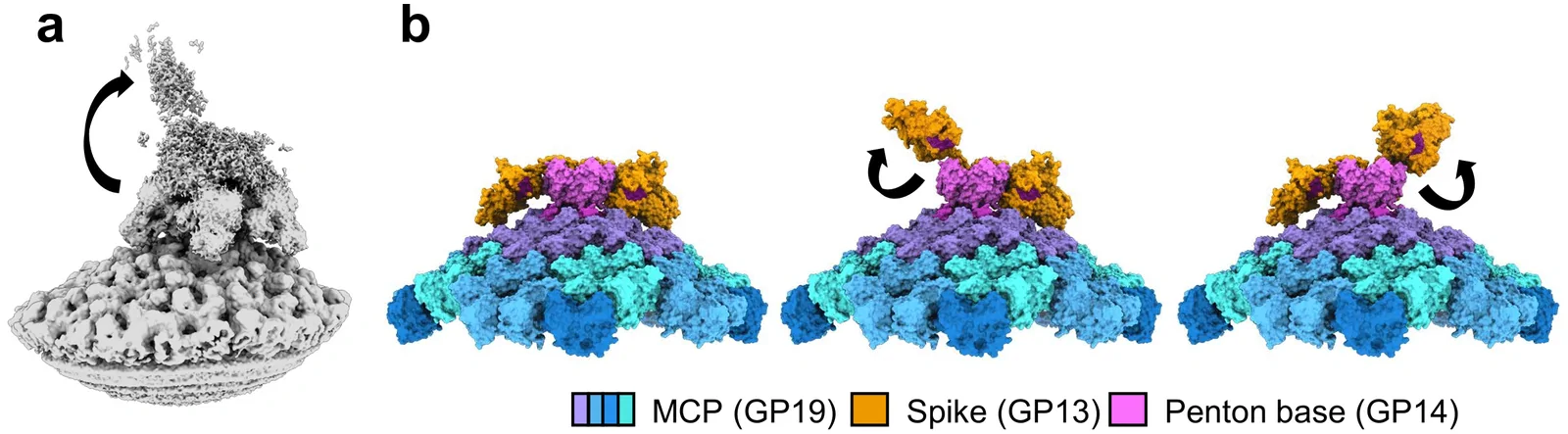
Spike mobility at the NO16 vertex. **(a)** Map obtained from localized reconstruction and classification showing density extending outwards from the penton, pointing to an open conformation of the spike. **(b)** Hypothetical model for GP13 spike mobility for receptor binding.

The spike protein GP13 presents a carbohydrate binding site and predicted glycosylase activity. Carbohydrate binding sites are also present in the spike protein forming the vertex turrets in archaeal DJR virus STIV [23]. It has been proposed that most viral proteins were recruited from cellular ancestral proteins, and indeed the β-sandwich structure is found in cellular proteins, most of which bind carbohydrates, and some are carbohydrate-active enzymes [40, 48]. The carbohydrate binding sites present in an ancestral cellular protein could have been maintained in the NO16 spike through exaptation, to play a role in host attachment and/or entry. A peptidoglycan sugar unit, *N*-acetyl-muramic acid, was identified as a receptor for bacteriophage Bam35, which infects Gram-positive bacteria [49]. The presence of three GP13 monomers in each NO16 spike would be advantageous by increasing the affinity for polysaccharides. Moreover, having two trimers located diametrically opposite to each other in each vertex may enhance the search space for host receptors. Additionally, blurry densities visualized on top of the vertex in some of the localized reconstruction classes (Fig 6a), might indicate mobility of the spikes, allowing exploration of the surrounding space and facilitating receptor binding (Fig 6b).

### A model for the complete NO16 infectious cycle

**Fig 7:**
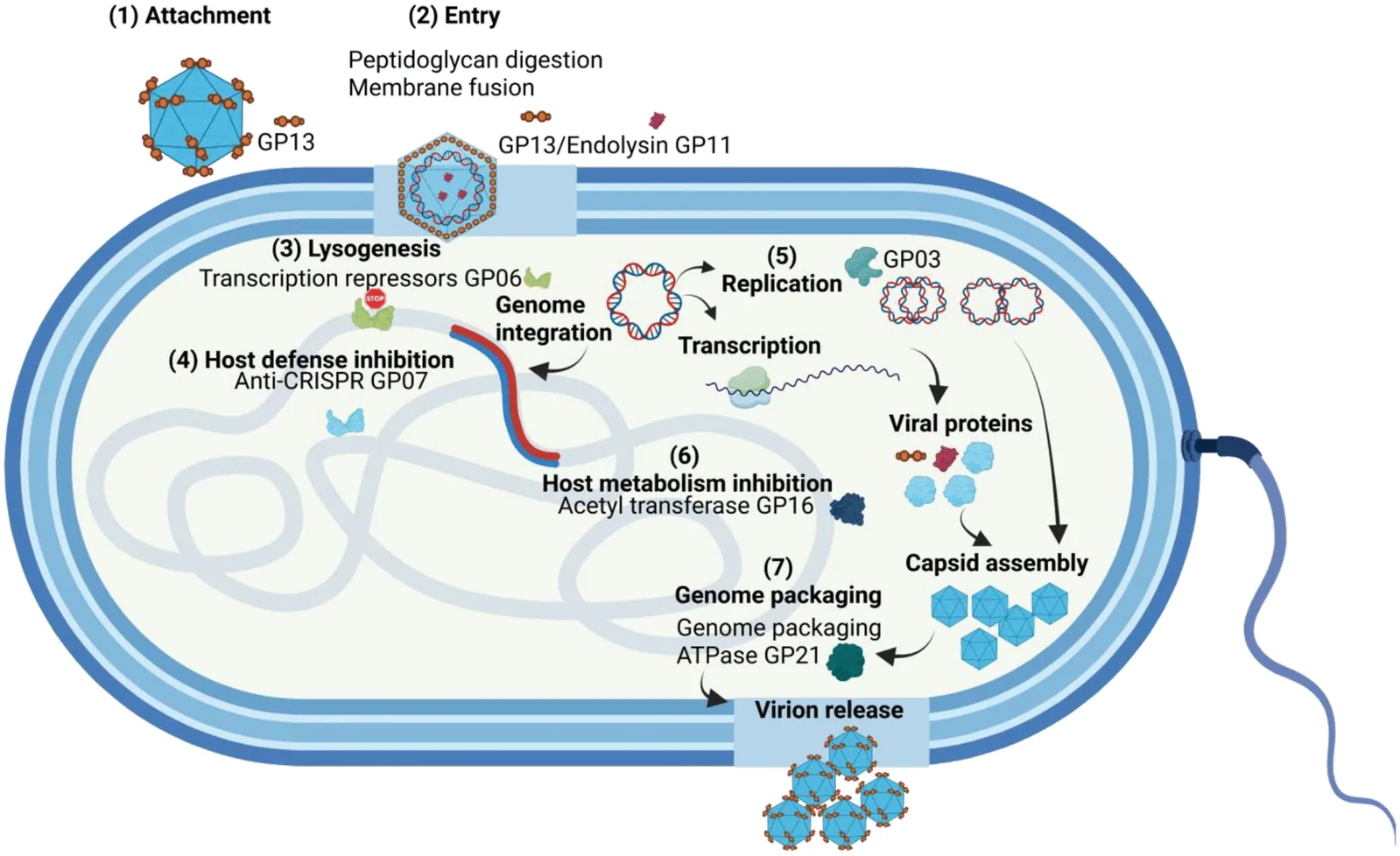
Hypothetical model for the NO16 infectious cycle. (1) First, NO16 attaches to the host cell wall via the GP13 carbohydrate binding site. (2) Entry occurs by carbohydrate degrading capacity of GP13 and (endo)lysin GP11, and most probably via membrane fusion. (3) Once the genome is released into the host cell, it integrates into the host genome and expression of transcription factor GP06 regulates the lytic/lysogenesis switch. (4) GP07 anti-CRISPR associated protein could inhibit host defence. (5) Replication via the rolling circle mechanism proceeds with participation of GP03. (6) GP16 acetyltransferase modulates host proteins by acetylating them. (7) The viral structural proteins assemble around host membranes, and the genome is packaged with the help of ATPase GP21. Created in BioRender. https://BioRender.com/gf4dr43.

Putting together the structure of the NO16 particle and the predictions for the non-traced proteins, we propose a hypothetical model for the NO16 infectious cycle (Fig 7). As the first step in infection, NO16 must cross the outer membrane, peptidoglycan layer, and inner membrane. Considering that vertex proteins usually mediate entry [30,31], the spike protein GP13, with its carbohydrate binding site and predicted glycosylase activity, could be involved in both attachment and entry (Fig 7, steps 1-2). Additionally, protein GP11, with predicted lysozyme activity, is present in the viral particle. Although endolysins have been associated mostly with the end of the replication cycle, the presence of this activity in the viral particle raises the intriguing possibility that GP11 could play a role in degradation of the peptidoglycan layer during NO16 entry (Fig 7, step 2). Bacteriophage Jorvik peptidoglycan hydrolase *Stl,* a homolog of GP11, has been shown to degrade peptidoglycan, supporting a similar lytic function for GP11 [7]. Similarly, bacteriophage PRD1 particles carry a membrane-associated transglycosylase that appears to play a role during the early stages of infection [50]. In related bacteriophages of the order *Vinavirales*, which include NO16 family *Asemoviridae, Autolykiviridae, Corticoviridae, Mestraviridae,* and *Parnassusviridae*, peptidoglycan hydrolyzers or endolysins are conserved [32]. We propose that GP13 and GP11 are potential candidates for lytic enzymes against *V. anguillarum* infections, which have a significant economic impact on fish farms [16].

Two entry mechanisms have been proposed for *Varidnaviria* bacteriophages: formation of a tube-like structure facilitating genome delivery in the linear-dsDNA phage PRD1 [51], and membrane fusion in the circular-dsDNA phage PM2 [21]. Since we did not observe any tube-like structure in the cryo-EM micrographs and the genome of NO16 is circular dsDNA as in PM2, we hypothesize that NO16 entry could occur via membrane fusion (Fig 7, step 2), similar to that proposed for PM2 [21].

Once inside the host cell, the NO16 genome integrates into the host genome through a site-specific recombination mechanism in a lysogenization process that is favoured at high host cell concentrations, suggesting that the lysis-lysogeny switch is likely regulated by host density-dependent mechanisms (e.g., quorum sensing) [17]. GP06 and GP07 expression was enhanced under conditions favoring lysogeny [17], supporting their role in regulating prophage induction/integration dynamics. Further, GP06 is a structural homolog of λ phage repressors that bind to lytic promoters [36] (Fig 7, step 4). Similarly, in non-tailed Jorvik phage, gp1 plays a role in lysis-lysogeny decision [7]. For GP07, we hypothesize an anti-CRISPR associated function, which could block bacterial defense systems against infections (Fig 7, step 4). A similar function has been attributed to STIV B116 protein [52].

Since GP03 is homologous to PM2 replication initiation protein and the NO16 genome is circular dsDNA, it seems likely that NO16 replication occurs via the rolling circle mechanism, with GP03 involved in the initiation process near the cytoplasmic membrane [53–55] (Fig 7, step 5). According to the structural homology of the replication proteins, the phage only codes for the replication initiation protein, and the other necessary proteins should be hijacked from the host. Mass spectrometry analysis of the purified virion revealed the presence of two host histone-like DNA-binding proteins (UniProt: A0A191W6X7 and A0A191W301) and the host DNA topoisomerase (UniProt: A0A1Y0NSD3), at higher abundance than some mCPs (S1b File). However, it remains unclear whether these proteins are components of the virion or were purified as contaminants. The replication mechanism of the circular dsDNA genomes in the DJR lineage is not well understood, as it is a different genome replication process than the common mechanism in the realm driven by the conserved type B DNA polymerase (PolB) [2, 56]. Notably, PolB is not encoded by any of the circular dsDNA or ssDNA non-tailed phages ΦCjT23, FLiP, PM2, and NO16. Moreover, the replication system components within bacteriophages of the *Vinavirales* order are frequently replaced [32], likely to adapt to different types of genome (circular ssDNA, linear dsDNA, or circular dsDNA).

GP16, a putative acetyltransferase, could alter host metabolism during infection (Fig 7, step 6) [57]. Acetyltransferases can neutralize protein charge by adding an acetyl group, and therefore, dramatically change protein function [57]. They also play a specific role in histone acetylation in eukaryotic cells [58]. Members of the GCN5-related *N-*acetyltransferases (GNAT) have been detected in multiple *Vinavirales* genomes, although not in PM2 [32]. Certain GNATs are associated with histone acetyltransferase activity [59]. There is recent evidence showing that bacteria encode proteins with hypothetical histone domains [60], and *V. anguillarum* proteins have been annotated as histone-like DNA-binding proteins (UniProt: A0A191W6X7 and A0A191W301). GP16 could, therefore, play a role in inhibiting bacterial host metabolism and/or host chromatin remodelling.

In the late stage of the infectious cycle, viral proteins assemble around host membranes into capsids, and the genome is incorporated with the help of genome packaging ATPase GP21 (Fig 7, step 7) [39]. Viral genomes are packaged into capsids by one of two mechanisms: concerted, where the protein shell is built around the genome [61–63]; or sequential, where the genome is pumped into a preformed empty capsid [64]. The genome packaging ATPase is conserved in the realm and plays a role in sequential packaging mechanisms [65] as well as in concerted packaging [61]. Therefore, the presence of a genome packaging ATPase in NO16 does not clarify the packaging mechanism.

In conclusion, this study provides new high-resolution data on the understudied *Varidnaviria* bacteriophages. This is the first structure of a non-tailed vibriophage, and of a phage in the newly defined *Asemoviridae* family. Notably, it also reveals the first symmetry-mismatched vertex complex solved at high resolution among viruses of the lineage (S1 Movie). To further complete the structural atlas of NO16, and considering that 70% of structural homologs that could be identified between viral and cellular proteins have no detectable sequence homology [66], we employed tertiary structure prediction tools and structure-based homology programs to assign the function of some viral proteins within the viral cycle. Although these findings offer valuable insights, further experimental validation is necessary to confirm the proposed infection model. Studying the structural and functional details of viruses infecting bacterial pathogens, such as *Vibrionaceae*, is essential for exploration of new therapeutic potentials.

## Materials and methods

### Virus and bacterial strains

Bacteriophage NO16 (GenBank: MH730557) and its host *Vibrio anguillarum* strain A023 (GenBank: JAHGUX000000000) were available in the Middelboe laboratory [15]. NO16 lysate was stored at 4°C, and host stock was stored at −80°C with 20% glycerol.

### Host culture

*V. anguillarum* was cultured in Marine Broth medium (MB; 0.5% tryptone, 0.1% yeast extract, and 2% sea salt, Himedia M1942-500G), incubated with agitation at 25°C. *V. anguillarum* colonies in MB 1.5% agar plates were stored at room temperature and inoculated into liquid medium for growth. Culture growth was monitored by measuring optical density at 600 nm wavelength (OD_600_).

### NO16 propagation and purification

*V. anguillarum* A023 culture was infected at OD_600_ = 0.15 (1 x 10^8^ cells/ml) with NO16 bacteriophage lysate at MOI = 0.1, and incubated at 25°C under agitation for approximately 3 hours until detection of complete lysis (OD_600_ ≤ 0.05). Cell debris was removed by centrifugation at 18,800 xg for 20 minutes at 4ºC, and the supernatant was filtered through 0.45 µm filters (Whatman, WHA10462600, cellulose acetate). The virus was concentrated by precipitation in 10% (w/v) PEG 8000 overnight at 4°C and diluted in PM2 buffer with magnesium (PM2-Mg buffer: 20 mM Tris-HCl pH 7.2, 100 mM NaCl, 5 mM CaCl_2_, 8 mM MgSO_4_ x 7 H_2_O) [19].

The bacteriophage was purified by ultracentrifugation in three subsequent gradients. First, the precipitated virus was loaded onto a linear 5-20% sucrose gradient diluted in PM2-Mg buffer for 1.5 hours at 24,000 rpm and 15°C (Beckman SW41 rotor). The pellet containing membranous contaminants was removed, and the upper fractions were concentrated again with 10% PEG 8000. The precipitate was loaded onto a linear gradient of 8-16% iodixanol diluted in PM2-Mg buffer (OptiPrep) with a 36% iodixanol cushion, and ultracentrifuged at 15°C, 30,000 rpm, for 35 minutes (Beckman SW55 rotor). The upper part of the cushion (approximately 1.5 ml) was loaded onto a 9-layer iodixanol gradient (21-24-27-30-33-36-42-48-54% iodixanol diluted in PM2-Mg buffer), and centrifuged for a minimum of 10 hours at 35,000 rpm and 18°C (Beckman SW41 rotor). The virus bands (running at approximately 30% iodixanol) were collected and stored at 4°C until further use. Prior to experiments, the purified NO16 virus sample was dialyzed against PM2-Mg buffer.

### Infectious titer determination

Double-layer agar assays were performed on M6 plates. For the bottom agar, 4 ml of MB medium with 1.5% agar were stirred in each well and left to harden. A volume of 1 ml of top agar (0.4% agar and 1% sea salt) was mixed with 100 µl of OD_600_ ~ 0.15 *V. anguillarum* culture and 10 µl of serial dilutions of the bacteriophage. Plates were stored at room temperature overnight, and lysis plaques were counted to estimate the number of plaque-forming units per milliliter (PFU/ml).

### Negative staining electron microscopy

To assess the homogeneity and structural integrity of purified virus samples, they were dialyzed against PM2-Mg buffer for one hour at 4°C, and imaged by negative staining electron microscopy. In-house collodion-carbon coated copper grids (Gilder G400-C3) were glow discharged for 15 seconds at 25 mA in a vacuum chamber to overcome the hydrophobicity of the carbon surface. Subsequently, samples were incubated on the grid for 5 minutes, stained for 30 seconds with 3 µL of 2% uranyl acetate, and blotted to remove excess liquid. Micrographs were obtained with a 120 kV JEOL JEM 1011 TEM at the CNB-CSIC Electron Microscopy Facility.

### Denaturing protein electrophoresis (SDS-PAGE)

Samples extracted from the gradients were mixed with 3x sample buffer (1% SDS, 1% β-mercaptoethanol, 10% glycerol, 50 mM Tris-HCl pH 6.8, 1.6% bromophenol blue) and heated at 95°C for 10 minutes. Proteins were separated by SDS-PAGE using 4-20% acrylamide gradient gels (Bio-Rad, 4568093). Electrophoresis was carried out for one hour at 90 V. After electrophoresis, gels were fixed for at least one hour (up to overnight) in 40% ethanol and 10% acetic acid, then sensitized in 30% ethanol, 125 mM sodium thiosulfate, and 800 mM sodium acetate for 30 minutes, and washed three times with Milli-Q water for 3 minutes. Afterwards, gels were stained with 0.25% silver nitrate for 20 minutes and washed twice for 30 seconds. Finally, gels were soaked in 240 mM sodium acetate and 0.015% formaldehyde until protein bands were distinguishable. The reaction was stopped by 40 mM ethylenediaminetetraacetic acid (EDTA) disodium salt dihydrate (EDTA-Na_2_ x 2H_2_O).

### Mass spectrometry

#### In gel digestion.

To identify the NO16 purification contaminants after SDS-PAGE, the band at 60 kDa was excised, cut into 1 mm^3^ pieces, and placed in a 96-well plate for automated trypsin digestion in an OT2 robot (Opentrons) as described elsewhere [67] with minor modifications. Briefly, gel pieces were first washed with 50 mM ammonium bicarbonate and then with acetonitrile (ACN) before simultaneous reduction and alkylation with 5 mM tris(2-carboxyethyl)phosphine hydrochloride (TCEP) and 10 mM chloroacetamide (CAA) in 50 mM ammonium bicarbonate. The gel pieces were then rinsed, first with 50 mM ammonium bicarbonate and then with ACN. Finally, they were dried at 40°C. Proteomics grade trypsin (Pierce) was added at a final concentration of 16 ng/μl in 25% ACN, 50 mM ammonium bicarbonate, and digestion was allowed to proceed at 37°C for 4 hours. Peptides were extracted by incubation with 50% ACN, 0.5% trifluoroacetic acid (TFA) for 25 minutes. They were then speed-vac dried and stored at −20°C until LC-MS/MS analyses.

#### Liquid S-Trap digestion.

The purified NO16 sample was diluted to 5% SDS and 25 mM triethylammonium bicarbonate (TEAB). The sample was reduced and alkylated by addition of 5 mM TCEP and 10 mM CAA for 30 minutes at 60ºC. Protein digestion in the S-Trap filter (Protifi, Huntington, NY, USA) was performed following the manufacturer’s instructions with minor changes [68]. The sample was digested at 37°C overnight using a trypsin:protein ratio of 1:15 and desalted with a StageTip C18 prior to analysis by LC-MS/MS.

#### LC-MS/MS Analysis.

Approximately 400 ng of peptides were dissolved in formic acid and subjected to LC-MS/MS analysis using an Ultimate 3000 nanoHPLC (Thermo Fisher Scientific) coupled online to an Orbitrap Exploris 240 mass spectrometer (Thermo Fisher Scientific). The HPLC was equipped with an Easy‐spray PepMap C18 analytical column (50 cm  ×  75 μm; Thermo Fisher Scientific). Solvents A and B were 0.1% formic acid and 0.1% formic acid in 80% acetonitrile, respectively. Peptides were separated with a 47 min gradient ranging from 4% to 95% B (60 minutes of total run time) at 50°C and a flow rate of 250 nl/min.

Data acquisition was performed using a data-dependent top 20 method in positive ionization mode. Survey scans were acquired at a resolution of 60,000 (FWHM). The top 20 most intense signals from each MS1 scan were selected for fragmentation by Higher-energy Collisional Dissociation (HCD) 30%. Resolution for MS2 spectra was set to 15,000 (FWHM). Precursor isolation was performed with a window of 1 m/z.

#### Database Search and Protein Identification.

Raw files were processed with Proteome Discoverer [69] (version 2.5.0.400; Thermo Fisher Scientific). MS2 spectra were searched with MASCOT [70] (version 2.8.0; Matrix Science), MSFragger [71], and Sequest against a database with the reference proteomes of bacteriophage NO16 (UniProt: UP000276974), its host, *Vibrio anguillarum* (UniProt: UP000197971), and a set of common contaminant sequences. Identifications were filtered at a false discovery rate (FDR) < 1% at the protein, peptide, and peptide-spectrum match (PSM) levels. Mass spectrometry analyses were carried out at the CNB-CSIC Proteomics Facility.

For the purified NO16 sample, copy numbers were estimated by dividing the abundance of each protein by that of GP19 (the MCP) and multiplying by the number of GP19 copies present in the virion. The result tables are included as S1 File. TTransmembrane predictions were obtained using TMHMM 2.0 (https://services.healthtech.dtu.dk/services/TMHMM-2.0/) [72].

### Cryo-EM sample preparation and data collection

Purified virus samples (1.5x10^11^ PFU/ml) were dialyzed against PM2-Mg buffer for one hour at 4°C and deposited in glow-discharged (1-minute, 25 mA) Quantifoil R1.2/1.3 Copper/Rhodium (Cu/Rh) grids. Three consecutive incubations on the grid with 3 µL drops of the sample followed by blotting were performed to improve the number of particles lying in the carbon holes [73]. Excess liquid was blotted, and grids were vitrified using an FEI Vitrobot. Grids were examined in a 200 kV Talos Arctica microscope at the CNB-CSIC cryo-EM facility to assess particle concentration, ice quality, and overall suitability of the sample. Cryo-EM data were collected at the Diamond Light Source Facility (eBIC, Oxford, United Kingdom) using a 300 kV Titan Krios microscope equipped with a Bioquantum K3 camera at a nominal pixel size of 1.34 Å at 64,000 x. Additional vitrification conditions and imaging parameters are summarized in S1 Table.

### Cryo-EM single particle averaging with icosahedral symmetry

All image processing was performed within the Scipion 3 framework [74]. Frame alignment was carried out using MotionCor2 [75]. The contrast transfer function (CTF) was estimated using CTFFIND4 [76]. Particles were semi-automatically picked using Xmipp3 [77] and extracted in 900 x 900 px boxes. 2D and 3D classifications were performed using RELION [78]. Icosahedral symmetry was enforced for 3D classification and refinement. The best 3D class was further processed by RELION 3D auto-refine, followed by CTF refinement [79]. The corrections were applied in the following order: beam tilt refinement, anisotropic magnification, and per-particle defocus correction. Finally, 3D auto-refine was run again, followed by resolution estimation (Fourier Shell Correlation (FSC) at 0.143 threshold) and high frequency boosting (B-factor, RELION post-process). The final map had a resolution of 3.3 Å (S2b Fig) [80]. Ewald sphere correction did not improve the resolution for this particular dataset, suggesting that other factors (e.g., number of particles, ice thickness, flexibility) may be limiting the resolution. Additional image processing, reconstruction, and refinement parameters are summarized in S1 Table. Local resolution was calculated with MonoRes [81] and depicted in ChimeraX.

### Localized reconstruction of vertex regions

Vertex subparticles centered on the 5-fold axis at the capsid radius were extracted with Localrec [82] in 320 x 320 px images (S5a Fig). Sixty subparticles were generated from each virus particle, and all downstream image processing was carried out without enforcing any symmetry. Due to uncertainty in the handedness convention followed by the software during subparticle extraction, the procedure was performed twice by changing the sign of the defocus correction. Both sets of subparticles were reconstructed in parallel, and the subset yielding higher resolution was selected for further processing. Next, extracted particles were realigned with CryoSPARC local refinement [83]. To enhance the signal of the five upper petals, we subtracted the capsid projection signal with RELION [78] in two stages. First, a mask based on the structure of the AU traced in the icosahedral map was used to obtain an initial model of the penton base GP14 distal domain and to properly fit the AlphaFold prediction of this domain. Second, a mask based on both the structure of the AU traced in the icosahedral map and the AlphaFold prediction of the penton base (GP14) distal domain was used to isolate the density corresponding to the five petals surrounding the penton base (S5b Fig). Subtracted particles were then classified into 10 classes using CryoSPARC (S5c Fig). The subparticles of the five classes showing at least one petal with high signal-to-noise ratio were selected and merged (S5c Fig, red box). To improve map resolution, we realigned the subparticles by rotating them by a multiple of 2П/5 rad to average the highest petal signal of every class (S5c Fig). The new pool of re-aligned subparticles was replaced by identical aligned subparticles without capsid subtraction for further RELION refinement. Redundant particles were removed before calculation of final maps (S5d Fig). Two refined maps were generated, either without using any mask, or using a localized mask encompassing the distal penton domain surrounded by two petals (S5d Fig), at 3.7 Å and 3.9 Å resolution, respectively. More details of the workflow are summarized in S5 Fig and in a *json* file (S3 File). Additional localized reconstruction maps were generated, focusing the refinement in one petal instead of at the 5-fold axis, using the workflow summarized in S7 Fig.

### Model building and analysis

Model building was completed within the Scipion 3 framework [84]. The initial models for GP19 and GP14 were predicted using AlphaFold 2 [85]. ChimeraX was used to perform a rigid fit of each initial model into the sharpened map [86]. Next, the fitted models were iteratively refined using Coot [87] and Phenix real-space refine [88]. To identify additional unmodeled densities, we generated remnant maps using ChimeraX by subtracting a map created from the traced model (*molmap* command) from the main cryo-EM map. ModelAngelo [89] was used to generate initial molecular models in the remnant map. In the first ModelAngelo search, no sequence was provided, and the results were submitted to BLAST (https://blast.ncbi.nlm.nih.gov/Blast.cgi) against the NO16 proteome. When hits had a significant e-value, a second ModelAngelo search was completed using the protein sequence of the hit. The resulting molecular models were refined using Coot and Phenix real-space refine.

Map and model visualization were carried out with ChimeraX [90]. The electrostatic surface potential was calculated using Adaptive Poisson Boltzman Solver (APBS) [91]. For structural alignment of the models, ChimeraX matchmaker was used [86]. Interactions between traced proteins were analyzed using PDBePISA [92]. Sequence alignments between NO16 GP18 and PM2 P3 were carried out with Clustal Omega [93], and visualized using ENDscript [94].

### Tertiary structure prediction, structural homology searches and function prediction

The structures of NO16 proteins not traced in the cryo-EM map were predicted using AlphaFold 3 (https://alphafoldserver.com/) [101]. The models were assessed based on the *predicted Template Modelling* (pTM) score, which evaluates the accuracy of a protein structure prediction. When multimers or additional cofactors were included in the prediction, the *interface predicted Template Modelling* (ipTM) score was used to assess the reliability of the interactions between subunits. A pTM score above 0.8 indicates high-confidence and high-quality predictions, whereas scores below 0.6 typically reflect failed predictions. If the initial prediction displayed a well-structured core with disordered C- or N-terminal regions, the disordered segments were removed, and the trimmed sequence was re-submitted to AlphaFold 3. Various multimerization states were also tested with AlphaFold 3.

Traced models, or predictions with considerable reliability (pTM > 0.75), were sent to DALI (PDB25 comparison) [25] and Foldseek (PDB100) servers [26] for structural homolog searches. Both results were considered to find a consensus. Even though DALI has been reported to be slower, it is more sensitive than Foldseek [95]. Additional sequence-based software tools used were: InterPro for classification of protein families [96], and Contrastive Learning enabled Enzyme Annotation (CLEAN) to predict Enzyme Commission (EC) numbers [97].

## Supporting information

S1 Table Cryo-EM vitrification conditions, data collection, image processing, reconstruction and refinement. (PDF)

S2 Table Modelling and validation statistics for NO16 molecular models and maps. Parameters provided by Phenix real-space refine [88].
(PDF)

S3 Table NO16 proteins traced in the model. In grey, chains traced in the localized reconstruction. (PDF)

S4 Table PDBePISA [92] analysis of NO16 capsid proteins. Chains colored by position in the asymmetric unit, according to the color legend in Fig 2c. The interface area (Å^2^) is calculated as half the difference between the total accessible surface areas of the isolated and interacting structures. ΔiG represents the solvation-free energy gain upon interface formation, and is calculated as the difference in total solvation energies between the isolated and interacting structures. A negative ΔiG indicates a favorable protein-protein interaction. N_HB_ indicates the number of potential hydrogen bonds formed across the interface. N_SB_ indicates the number of potential salt bridges across the interface. N_DS_ indicates the number of disulfide bonds across the interface. Notice that, since PDBePISA considers interactions between atoms, some of the salt bridges may include several atoms from the same residue.
(PDF)

S5 Table PDBePISA analysis of PM2, FLIP and ØCjT23 MCP capsomers. For comparison with S4 Table. Parameters are described in S4 Table.
(PDF)

S6 Table Structure prediction and homology search results for all proteins coded by the NO16 genome. MS, mass spectrometry. P, present in the virion in mass spectrometry analysis. pTM, predicted template modelling score for AlphaFold predictions. T, traced protein. IDR, intrinsically disordered region. TM, transmembrane. EC, Enzyme Commission number. Z-scores for homology search using DALI in the range from 8 to 20 indicate probable homology; values above 20 indicate definite homology; scores below 2 are considered nonsignificant. For Foldseek, E-values equal or lower than 0.01 were considered. Proteins for which a likely function has been assigned are highlighted in green.
(PDF)

S1 Fig AlphaFold 3 predictions of NO16 proteins detected in purified virions by mass spectrometry. Models are colored by prediction confidence. The pTM score is indicated next to each model.
(TIF)

S2 Fig NO16 cryo-EM data, average and local resolution. (**a**) Cryo-EM motion corrected micrograph (left), and representative NO16 particles (centre and right). The scale bar represents 100 nm in the micrograph and 20 nm on the particles. (**b**) Fourier Shell Correlation (FSC) curves indicating an average resolution of 3.3 Å in the final map as provided by RELION post-process. Note that the corrected and masked map curves overlap. (**c**) Cryo-EM map colored by local resolution, calculated by Xmipp Local MonoRes [81].
(TIF)

S3 Fig Comparison of the MCP structure of *p*T = 21 bacteriophages in the DJR lineage. (**a**) Monomers of NO16, PM2 (PDB: 2vvf), FLiP (PDB: 5oac), and ΦCjT23 (PDB: 7zzz) MCPs with α-helices in pink and β-sheets in blue. Protein names and length in amino acids (aa) are indicated. View from the intra-capsomer side. (**b**) Structural similarity dendrogram derived from DALI Z-scores in the all-against-all comparison. **(c)** Structural similarity heatmap based on DALI Z-scores. (**d**) Pairwise structural superpositions generated using the matchmaker tool in ChimeraX. Colors are indicated in the legend. (**e**) MCP monomers colored by surface electrostatic potential, oriented as in (a). (**f**) Cation coordination site at the base of the NO16 MCP visualized in ChimeraX. Solid circles indicate the cation map density, and the dashed circle denotes its absence. Residues involved in the interactions are labeled. Maps are contoured at 4.5σ.
(TIF)

S4 Fig Effect of chelating agents on NO16 infectivity, and ion identity prediction using the *Metric Ion Classification* (MIC) tool. (**a**) NO16 viral titer quantified after 24 hours of treatment with different concentrations of EDTA or EGTA. We dialyzed NO16 lysate against PM2-Mg buffer (control), or against the same buffer with 20 mM or 100 mM concentrations of EDTA or EGTA. After 24 hours at 4°C, viral titers were determined by double-layer agar assay in biological triplicates. We found no statistically significant differences in infectivity across the tested conditions, although a slight reduction in titer was observed in all treated samples. (**b**) MIC [99] confidence estimations for MCP-bound ions or water in NO16 and PM2. Either Na⁺ or water were predicted for both NO16 and PM2, mostly with confidence below the recommended limit (>0.7, for a 0-1 range).
(TIF)

S5 Fig Localized reconstruction workflow of NO16 vertex region. **(a)** Extraction and alignment of vertex subparticles from the icosahedral refined map centered at the 5-fold symmetry axis. (**b**) First stage of capsid projection subtraction in the aligned particles to model automatically the distal domain of GP14. This initial GP14 model allows matching the AlphaFold structure prediction to the GP14 distal domain. (**c**) Second stage of capsid projection subtraction in the aligned particles to remove all capsid signal except the five petals surrounding the penton base. Classification on CryoSPARC2 without alignment of subtracted particles discriminates the selected oriented pool of particles for further refinement. (**d**) Assignment of subtracted particle orientations to the aligned initial subparticles, to perform the local refinement of the whole vertex (without masking) and of the distal domain of penton base surrounded by two opposing petals (with local masking). Top views show slice 180 of the reconstructed map; side views show the central slice 160. The entire processing was performed using the Scipion software framework with methods from plugins described in https://scipion.i2pc.es/report_protocols/packages. (1) Scipion [74]. (2) Scipion for modelling [84]. (3) RELION [78]. (4) Localrec [82]. (5) CryoSPARC2 [83]. (6) ChimeraX [86]. (7) Xmipp3 [100]. (8) ModelAngelo [89]. (9) AlphaFold 3 [101].
(TIF)

S6 Fig Localized reconstruction of the NO16 vertex region. **(a)** Fourier Shell Correlation (FSC) curves for the localized reconstruction, showing an average resolution of 3.9 Å, as provided by RELION post-process. (**b**) NO16 localized reconstruction colored by local resolution, calculated by Xmipp Local Monores. (**c**) Molecular model and remnant densities in the localized reconstruction. Remnant densities correspond to regions in the map where density is present, but the identity of the amino acids could not be unequivocally assigned. (**d**) Map obtained from localized reconstruction and classification with one spike fixed at position 1, showing weak density for opposing spike positions 3 and 4.
(TIF)

S7 Fig Workflow for inspecting adjacent positions of a fixed spike in the NO16 vertex region. **(a)** Extraction and alignment of vertex subparticles from the icosahedral refined map centered on a vertex spike. (**b**) Capsid projection subtraction in the aligned particles to remove any capsid signal except the five petals surrounding the penton base. Classification without alignment of subtracted particles discriminates between presence and absence of spike in position 1. Both subparticle classes generated are selected for further refinement. (**c, d**) Assignment of subtracted particle orientations to the aligned initial subparticles to perform the local refinement of the whole vertex. The refined orientations of subparticles will be assigned again to subtracted particles to locally align the distal penton base and surrounding spikes. Top views show slice 154 of the reconstructed map while side views show slice 200. The entire processing was performed using the Scipion software framework using methods from plugins described in https://scipion.i2pc.es/report_protocols/packages. (1) Scipion [74]. (2) Scipion for modelling [84]. (3) RELION [78]. (4) Localrec [82]. (5) CryoSPARC2 [83]. (6) ChimeraX [88]. (7) Xmipp3 [100].
(TIF)

S8 Fig Comparison of the NO16 map with those of other *pT* = 21 bacteriophages. Central slices of cryo-EM maps for NO16 (this work), PM2 (EMDB:1082), ΦCjT23 (EMDB:15042) and FLiP (EMDB: 3771). C, capsid; L; lipid membrane. The scale bar represents 20 nm. The rounded rectangle indicates capsid-membrane connections.
(TIF)

S9 Fig Comparison between NO16 and PM2 minor capsid proteins. (**a**) Asymmetric unit (AU) seen from the inside of the capsid, colored by surface electrostatic potential. Left, AU with mCPs removed; their positions are indicated by dashed silhouette outlines. Right, mCP surfaces interacting with MCP capsomers. (**b**) Schematic organization of PM2 P3 and P6 mCPs, depicted on the NO16 facet. (**c**) Capsomers 2 and 4 seen from inside the capsid (left) and from the side (right). NO16 GP18 protein and PM2 P3 overlap. Red arrows indicate the conformation differences of the GP18 C-terminal region compared to PM2 P3. Right, molecular models of GP18 chains M and N, along with their cryo-EM density. (**d**) Localized reconstruction map showing remnant densities, with traced NO16 and PM2 mCPs represented as cartoons.
(TIF)

S10 Fig AlphaFold 3 predictions for NO16 encoded proteins not detected in the virion. See S1 Fig for proteins detected in the viral particle by mass spectrometry. Models are colored by prediction confidence. pTM and ipTM values (for oligomers) are indicated next to each model. Red arrows indicate the helicase domain of GP21.
(TIF)

S1 File Mass spectrometry results of NO16: (a) 60 kDa contaminant band. (b) purified NO16 virion. (XLSX)

S2 File Structural homology search results of NO16 proteins. (XLSX)

S3 File Localized reconstruction workflow. (JSON)

S1 Movie Summary of NO16 structure. (MP4)

S1 Raw Gel Silver stained SDS-PAGE analysis of NO16 particles purified by the optimized protocol. Raw image for Fig 1c.
(PDF)
