# Supplementary material for: Structure of NO16, a marine non-tailed vibriophage with an unusual symmetry-mismatched vertex arrangement
Source: PLoS Pathog. 2026 Sep 8;22(9):e1014525. doi: 10.1371/journal.ppat.1014525 (PMC13552765; doi:10.1371/journal.ppat.1014525)
Supplement: Supplementary file 1 [file ppat.1014525.s001.pdf]

Table S1. Cryo-EM vitrification conditions, data collection, image processing, reconstruction and refinement.

**Vitrification**		
Grids	Quantifoil R1.2/1.3 Cu/Rh	
Glow discharge	25 mA, 1 minute	
Temperature	22ºC	
Humidity	95%	
Blot time	3 seconds	
Force	- 4	
**Data collection**		
Microscope	Titan Krios	
Camera	Bioquantum K3	
Voltage	300 kV	
Magnification	64,000 x	
Nominal pixel size	1.34 Å/px	
Exposure time	4.66 s	
Total dose rate	39.97 e-/Å^2	
Number of frames	40	
Defocus range	-1.1 to -2.5 μm, in -0.2 μm steps	
Micrographs collected	20,640	
Acquisition software	EPU 3.5.1	
**Image processing**		
Frame alignment software	MotionCor2, Scipion 3	
CTF estimation software	CTFFIND4, Scipion 3	
Particle picking software	Xmipp3, Scipion 3	
Micrographs used	20,526	
Particles picked	18,657	
**Reconstruction**	**Whole capsid**	**Localized reconstruction**
Software	RELION, Scipion 3	RELION, CryoSPARC
Particles included	15,429	162,312 (subparticles)
Symmetry imposed	Icosahedral	C1
Rotational accuracy	0.029 degrees	1.89 degrees
Translational accuracy	0.1005 pixels	1.24 pixels
B-factor applied	-108.24 Å^2	-131.28 Å^2
Final resolution (gold standard FSC = 0.143)	3.3 Å	3.9 Å
