# Supplementary material for: Structure of NO16, a marine non-tailed vibriophage with an unusual symmetry-mismatched vertex arrangement
Source: PLoS Pathog. 2026 Sep 8;22(9):e1014525. doi: 10.1371/journal.ppat.1014525 (PMC13552765; doi:10.1371/journal.ppat.1014525)
Supplement: Supplementary file 2 [file ppat.1014525.s002.pdf]

Table S2. Modelling and validation statistics for NO16 molecular models and maps. Parameters provided by Phenix real-space refine [1].

	**Icosahedral map**	**Localized reconstruction**
Software	Scipion, ModelAngelo, ChimeraX, Coot, Phenix	
Model	PDB ID 9T9V	PDB ID 9T9R
Chains	19	8
Atoms	22065 (Hydrogens: 0)	6877 (Hydrogens: 0)
Residues	Protein: 2864 Nucleotide: 0	Protein: 893 Nucleotide: 0
Water	0	0
Ligands	4	0
Bond length (RMSD, Å)	0.004	0.007
Bond angles (RMSD, °)	0.97	1.205
MolProbity score	1.73	2.35
Clash score	7.62	17.12
**Ramachandran plot (%)**		
Outliers	0.04	0.36
Allowed	4.41	12.46
Favoured	95.56	87.19
**Rama-Z (Ramachandran plot Z-score, RMSD)**		
whole	(N= 2834) -0.98 (0.16)	(N= 835) -2.96 (0.27)
helix	(N= 405) 1.13 (0.28)	(N= 18) -3.03 (1.09)
sheet	(N= 998) -0.58 (0.17)	(N= 179) -0.98 (0.43)
loop	(N= 1432) -1.19 (0.16)	(N= 638) -2.44 (0.22)
Rotamer outliers (%)	0.17	0.77
Cβ outliers (%)	0	0
