# Supplementary material for: Structure of NO16, a marine non-tailed vibriophage with an unusual symmetry-mismatched vertex arrangement
Source: PLoS Pathog. 2026 Sep 8;22(9):e1014525. doi: 10.1371/journal.ppat.1014525 (PMC13552765; doi:10.1371/journal.ppat.1014525)
Supplement: Supplementary file 3 [file ppat.1014525.s003.pdf]

Table S3. NO16 proteins traced in the model. In grey, chains traced in the localized reconstruction.

Protein	Length (amino acids)	Copy number in the AU*	Chain ID	Residues traced	Not traced
**Icosahedral AU**					
**MCP (GP19)**	265	10	A	2-265	1
			B	2-265	1
			C	2-265	1
			D	2-265	1
			E	2-265	1
			F	2-265	1
			G	2-265	1
			H	2-265	1
			I	2-265	1
			J	2-265	1
**Penton base proximal domain (GP14) 1-88 aa**	180	1	K	2-88	Distal domain traced in PDB vertex
**Minor capsid protein (GP18)**	82	3	L	4-50	1-3; 51-82
			M	9-42	1-8; 43-82
			N	2-55	1; 56-82
**Unknown**	-	1	O	1-12 (UNK)	-
**Non-icosahedral vertex**					
**Penton base distal domain (GP14) 89-180aa**	180	1	A	66-69, 89-180	Proximal domain in PDB AU
			B	89-131,143-180	132-142
			C	89-99,104-131, 143-149, 159-180	100-103,132-142,150-158
			D	89-101, 108-130,141-163,172-180	102-107,131-140,164-171
			E	89-101,110-129,139-152, 171-180	102-109,130-138,153-170
**Spike (GP13)**	235	SM**	F	11-235	1-10
		SM	G	8-36,45-60,81-88, 137-225, 230-235	1-8,37-44,61-80,89-136,226-229
		SM	H	7-36, 45-59, 80-87, 136-225, 230-235	1-6,37-44,60-79,88-135,226-229

*AU: icosahedral asymmetric unit. **SM: Symmetry mismatch.
