# Supplementary material for: Structure of NO16, a marine non-tailed vibriophage with an unusual symmetry-mismatched vertex arrangement
Source: PLoS Pathog. 2026 Sep 8;22(9):e1014525. doi: 10.1371/journal.ppat.1014525 (PMC13552765; doi:10.1371/journal.ppat.1014525)
Supplement: Supplementary file 4 [file ppat.1014525.s004.pdf]

Table S4. PDBePISA [2] analysis of NO16 capsid proteins. Chains colored by position in the asymmetric unit, according to the color legend in Fig. 2c. The interface area (Å^2) is calculated as half the difference between the total accessible surface areas of the isolated and interacting structures. ΔiG represents the solvation-free energy gain upon interface formation, and is calculated as the difference in total solvation energies between the isolated and interacting structures. A negative ΔiG indicates a favorable protein-protein interaction. N_{HB} indicates the number of potential hydrogen bonds formed across the interface. N_{SB} indicates the number of potential salt bridges across the interface. N_{DS} indicates the number of disulfide bounds across the interface. Notice that, since PDBePISA considers interactions between atoms, some of the salt bridges may include several atoms from the same residue.

**Chain 1 (capsomer position)**	**Chain 2 (capsomer position)**	**Interface area (Å^2)**	**ΔiG (kcal/mol)**	**N_{HB}**	**N_{SB}**	**N_{DS}**
Intra-capsomer contacts between MCP						
J (4)	H (4)	852.4	-2.3	4	11	0
F (2)	D (2)	836.9	-1.0	5	12	0
F (2)	E (2)	836.4	-4.4	4	9	0
E (2)	D (2)	818.1	-4.4	6	10	0
J (4)	I (4)	816.3	-3.9	8	11	0
I (4)	H (4)	815.8	-2.9	5	9	0
B (1)	A (1)	796.0	-3.5	2	6	0
C (1)	B (1)	783.8	-2.0	6	8	0
C (1)	A (1)	636.7	2.2	2	8	0
Inter-capsomer contacts between MCP						
H (4)	C (1)	584.9	-1.0	5	1	0
I (4)	F (2)	425.9	-1.2	1	1	0
I (4)	G (3)	395.5	-2.0	0	0	0
D (2)	B (1)	389.4	-4.0	0	0	0
G (3)	F (2)	378.3	-0.4	0	2	0
I (4)	C (1)	230.0	0.7	1	0	0
I (4)	D (2)	159.4	1.1	1	0	0
D (2)	C (1)	156.9	1.2	0	0	0
J (4)	G (3)	137.6	0.3	2	0	0
Contacts between MCP-penton						
K	A (1)	719.2	-2.5	2	5	0
Contacts between spike-penton						
F (GP13)*	A (GP14)*	590.7	-6.0	4	0	0
Contacts between MCP-mCP						
N	J (4)	1408.8	-19.3	7	2	0
L	C (1)	1311.7	-14.8	6	2	0
M	D (2)	961.1	-15.2	2	1	0
M	F (2)	346.6	-0.6	2	6	0
N	I (4)	327.4	-0.9	4	4	0
L	B (1)	317.7	-1.7	0	3	0
N	G (3)	314.0	0.7	2	0	0
L	D (2)	173.2	-2.0	0	0	0
N	H (4)	210.9	-4.7	1	0	0
L	A (1)	196.6	-2.9	1	0	0
O	H (4)	197.5	-3.7	1	0	0
L	I (4)	197.2	-0.2	5	0	0
O	I (4)	149.0	-2.9	0	0	0
M	I (4)	95.2	-0.7	0	0	0
O	C (1)	26.3	-0.5	0	0	0

*From the non-icosahedral vertex molecular model.
