# Supplementary material for: Structure of NO16, a marine non-tailed vibriophage with an unusual symmetry-mismatched vertex arrangement
Source: PLoS Pathog. 2026 Sep 8;22(9):e1014525. doi: 10.1371/journal.ppat.1014525 (PMC13552765; doi:10.1371/journal.ppat.1014525)
Supplement: Supplementary file 5 [file ppat.1014525.s005.pdf]

Table S5. PDBePISA analysis of PM2, FLIP and ØCJT23 MCP capsomers. For comparison with Table S4. Parameters are described in Table S4.

	Chain 1	Chain 2	Interface area (Å^2)	Δ^iG (kcal/mol)	N_{HB}	N_{SB}	N_{DS}
**PM2**	A	B	1164.9	-6.7	21	10	0
	B	C	1159.1	-7.7	21	10	0
	A	C	1153.8	-7.5	21	10	0

	Chain 1	Chain 2	Interface area (Å^2)	Δ^iG (kcal /mol)	N_{HB}	N_{SB}	N_{DS}
**FLIP**	A	B	1423.4	-8.5	6	3	0
	B	C	1381.8	-8.9	6	3	0
	A	C	1381.5	-8.9	10	5	0

	Chain 1	Chain 2	Interface area (Å^2)	Δ^iG (kcal /mol)	N_{HB}	N_{SB}	N_{DS}
**ØCJT23**	A	B	913.7	-8.2	8	5	0
	B	C	913.5	-8.9	10	6	0
	A	C	883.1	-9.4	10	5	0
