# Supplementary material for: Structure of NO16, a marine non-tailed vibriophage with an unusual symmetry-mismatched vertex arrangement
Source: PLoS Pathog. 2026 Sep 8;22(9):e1014525. doi: 10.1371/journal.ppat.1014525 (PMC13552765; doi:10.1371/journal.ppat.1014525)
Supplement: Supplementary file 6 [file ppat.1014525.s006.pdf]

Table S6. Structure prediction and homology search results for all proteins coded by the NO16 genome. MS, mass spectrometry. P, present in the virion in mass spectrometry analysis. pTM, predicted template modelling score for AlphaFold predictions. T, traced protein. IDR, intrinsically disordered region. TM, transmembrane. EC, Enzyme Commission number. Z-scores for homology search using DALI in the range from 8 to 20 indicate probable homology; values above 20 indicate definite homology; scores below 2 are considered nonsignificant. For Foldseek, E-values equal or lower than 0.01 were considered. Proteins for which a likely function has been assigned are highlighted in green.

**Protein**	**Previous annotation** (if not specified, unknown)	**MS**	**New annotation**	**pTM**	**Structural based search for homologues**		**Sequence based software**	
					**DALI (PDB25)**	**Foldseek (PDB100)**	**InterPro**	**CLEAN**
**GP01**				0.72	-	-	n/a	Fumarate hydratase EC:4.2.1.2 (low)
**GP02**				0.8	<2	>0.01	n/a	Methyltransferase EC:2.1.1.86 (low)
**GP03**			Replication initiation protein	0.78	SIRV1 hypothetical protein ORF119, Replication initiation protein (PDB: 2x3g, Z-score: 9.3)	>0.01	340-409 aa Replication-associated protein ORF2/G2P (IPR056906)	DNA ligase (ATP) EC:6.5.1.1 (low)
**GP04**				0.26	-	-	n/a	Deoxyribonuclease EC:3.1.21.4 (low)
**GP05**				0.39	-	-	n/a	Decarboxylase EC:4.1.1.44 (low)
**GP06**	Putative DNA-binding protein		Lytic and lysogenic transcriptional regulator	0.84	HTH-type transcription regulators (PDB: 2eby, Z-score: 7.7)	ImmR transcriptional regulator DNA-binding domain of *Bacillus subtilis* (PDB: 7t8i, E-value:5.17e-2)	n/a	Transferring alkyl or aryl groups EC:2.5.1.83 (low)
**GP07**	S-adenosylhomocysteine protein		Anti-CRISPR-associated protein	0.49	Anti-CRISPR-associated Aca2 (PDB: 7ezy, Z-score: 10)	Anti-CRISPR-associated protein Aca2 (PDB: 7vjq, E-value: 1.80e-1)	55-111 aa Phage protein (IPR021077)	Methyltransferases EC:2.1.1.148 (low)
**GP08**		P		0.49	-	-	22-41 aa TM	Methyltransferases EC:2.1.1.86 (low)
**GP09**				0.38	-	-		Methyltransferases EC:2.1.1.86 (low)
**GP10**	Structurally similar to PM2 protein p6	P		0.47	-	-		Thiosulfate dehydrogenase EC:1.8.5.2 (low)
**GP11**	Protein inside capsid D	P	Endolysin	0.93	Endolysin from bacteriophage Enc34 (PDB: 7q47, Z-score: 17.4)	Endolysin from bacteriophage Enc34 (PDB: 7q47, E-value: 1.712e-09)	1-20 aa IDR	Lysozyme EC:3.2.1.17 (low)

Protein	Previous annotation (if not specified, unknown)	MS	New annotation	pTM	Structural based search for homologues		Sequence based software	
					DALI (PDB25)	Foldseek (PDB100)	InterPro	CLEAN
**GP12**				0.64	-	-	40-64 aa IDR	Peptidase EC:3.4.22.65 (low)
**GP13**		P	Peptidoglycan hydrolase	T	S-layer associated multidomain endoglucanase (PDB: 2zew, Z-score: 9.6)	Vip3Bc1 and Vip3Aa tetramer (PDB: 6yrf/6tfk, E-value: 2.29e-02, 4.63e-02)	n/a	Glycosylases EC:3.2.2.22 (low)
**GP14**		P	Penton base protein	T			n/a	Carbon-carbon bonds ligase EC:6.4.1.8 (low)
**GP15**				0.36	-	-	128-145 aa TM	Methyltransferases EC:2.1.1.86 (low)
**GP16**		P	Acetyl transferase	0.87	Iaa acetyltransferase (PDB: 1y9k, Z-score: 13)	*Bacillis subtilis* Acetyltransferase (PDB: 1yvk, E-value:2.73e-6)	n/a	Acid-thiol ligases EC:6.2.1.14 (low)
**GP17**				0.31	-	-	1-35 aa IDR, 49-66 aa TM	Carboxy- and carbamoyltransferases EC:2.1.3.1 (low)
**GP18**	Structurally similar to PM2 protein p3	P	Minor capsid protein	T	-	-		Glycosylases EC:3.2.1.39 (low)
**GP19**	Major capsid protein - DJR viral lineage	P		T	-	-	2-134 aa, 135-264 aa Viral major capsid protein (IPR056906)	Glycosylases EC:3.2.1.129 (low)
**GP20**				0.42	-	-	n/a	Oxidoreductases EC:1.12.2.1 (low)
**GP21**	Putative ATPase-DNA translocase ftsk			0.93	NTPase from STIV2 (PDB: 4kfs, Z-score of 19.3)	NTPase from STIV2 (PBD:4kfr, and E-value of 1.74e-12)	12-90 aa Helicase HerA, central domain;113-188 aa Helicase HerA-like (IPR008571)	Site-specific deoxyribonuclease EC:3.1.21.4 (low)
**GP22**				0.45	-	-	n/a	Transferase (amino group) EC:5.4.3.3 (low)
**GP23**		P		0.55	-	-	n/a	Decarboxylase EC:7.2.4.3 (low)
