# Supplementary material for: Structure of NO16, a marine non-tailed vibriophage with an unusual symmetry-mismatched vertex arrangement
Source: PLoS Pathog. 2026 Sep 8;22(9):e1014525. doi: 10.1371/journal.ppat.1014525 (PMC13552765; doi:10.1371/journal.ppat.1014525)

Silver stained SDS-PAGE analysis of NO16 particles purified by the optimized protocol. Raw image for Figure 1c.

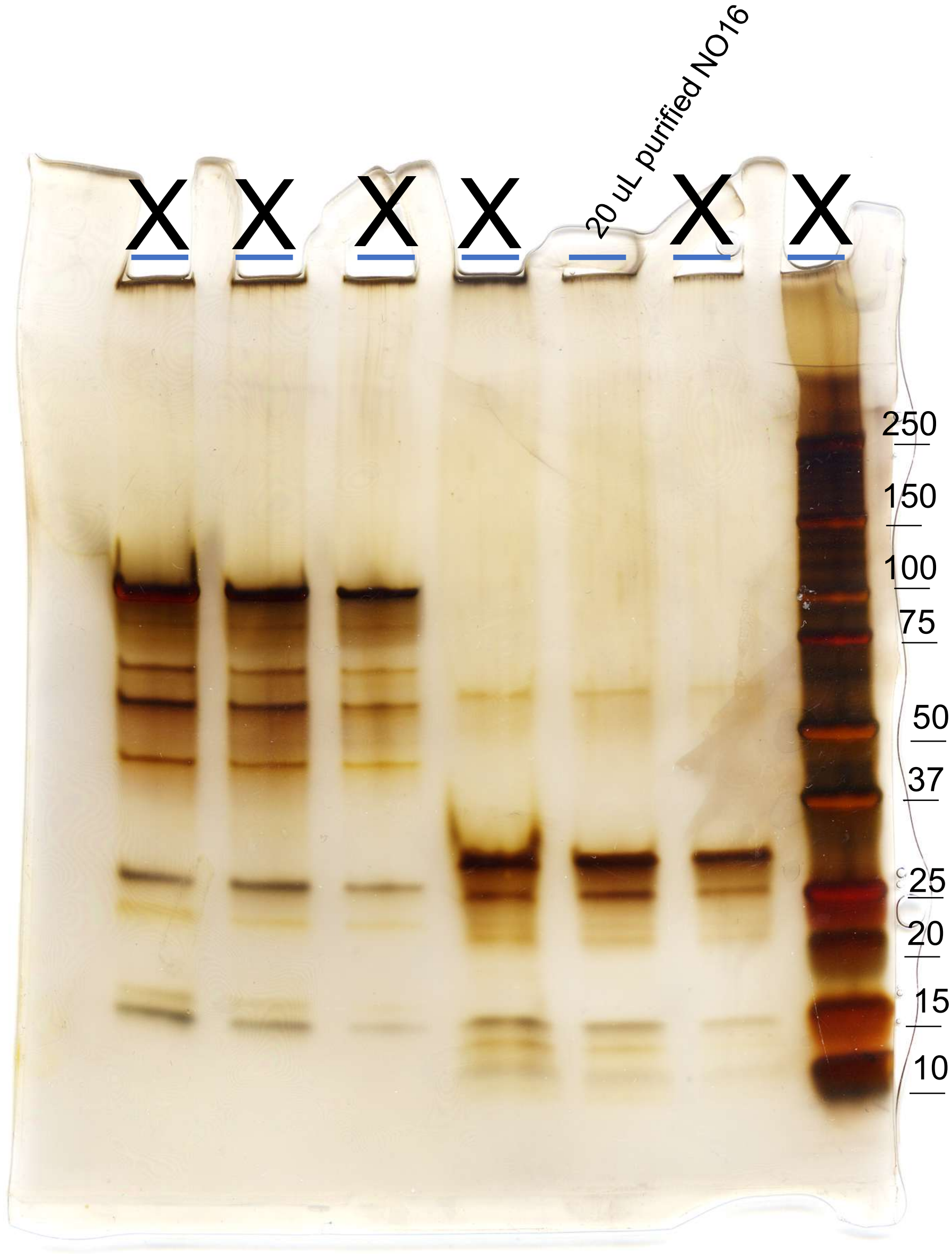

Supplement: Supplementary file 7 [file ppat.1014525.s021.pdf]
